# Development of Lower-Limb Power Across Age and Sex in Junior and Elite Artistic Gymnasts

**DOI:** 10.3390/jfmk11010096

**Published:** 2026-02-26

**Authors:** Christoph Schärer, Fabio Strauss, Nubya Baio, Caterina Barloggio, Anne-Sarah Dysli

**Affiliations:** 1 Swiss Federal Institute of Sport Magglingen (SFISM), 2532 Magglingen, Switzerland; 2 Swiss Gymnastics Federation (STV), 5000 Aarau, Switzerland

**Keywords:** lower extremities, physical requirements, explosive strength, reactive strength

## Abstract

**Background**: Explosive and reactive strength of the lower extremities are essential performance determinants in artistic gymnastics. This study analyzed ten years of performance diagnostic data from Swiss junior and elite gymnasts to describe age- and sex-specific development patterns of lower-limb strength and to examine whether early strength levels predict later selection to the Swiss national elite team. **Methods**: Longitudinal performance data from 234 Swiss gymnasts (125 females, 109 males; 7–30 years) were analyzed. Explosive and reactive strength were assessed using countermovement, squat, single-leg, and drop jumps. Age- and sex-related differences, as well as associations with later national team selection, were examined. **Results**: Explosive and reactive strength increased significantly across age categories in both sexes (*p* < 0.001). In females, the greatest improvements occurred between U14 and U16 (explosive strength: +8.7–12.9%; reactive strength: +15.6–21.2%), followed by stagnation in explosive strength at older ages. In males, both strength qualities increased continuously from U14 to U18 (+9.3–15.7% per category; *p* < 0.001), with smaller gains at the elite level. Sex differences in explosive strength emerged from U16 and became pronounced from U18 onwards, favoring males (+11.3–19.2%). Reactive strength showed smaller and partly reversed sex differences, with females demonstrating higher values in U16 and elite athletes (up to +16.5%). Differences between athletes later selected for the national team and non-selected athletes were small and mostly non-significant. **Conclusions**: Lower-limb strength development in gymnastics follows distinct age- and sex-specific patterns. Systematic training appears to moderate sex-related differences, particularly in reactive strength. While physical capacities form an important foundation, technical proficiency seems to play a more decisive role in elite selection and long-term athlete development.

## 1. Introduction

Women’s and men’s artistic gymnastics are technically complex and physically demanding sports with a long historical tradition. Over recent decades, continuous developments in apparatus design, competition formats, scoring systems, and athletes’ technical and physical performance levels have shaped the sport [1]. Improvements in the spring properties of the apparatus may have facilitated higher element difficulty and altered the physical demands placed on athletes, thereby increasing the importance of physical attributes—particularly strength and power capacities—as fundamental prerequisites for successful performance in both disciplines [2]. Among these attributes, the explosive and reactive strength of the lower extremities are key determinants of performance on apparatus, such as the floor, vault, and, to some extent, the balance beam [2,3,4,5,6]. In this context, it should be noted that lower-limb power represents a key performance prerequisite for women on three of their four competition apparatus, whereas in men’s artistic gymnastics, decisive lower-limb physical demands are primarily required on only two of the six apparatus. The growing difficulty of acrobatic elements and the higher complexity of movement sequences introduced in recent years have further elevated the importance of these qualities [2,7]. Gymnasts rely on explosive and reactive power to generate high approach velocities, produce powerful take-offs within short ground contact times, and achieve precise and controlled landings [8,9]. These capabilities not only determine jump height and flight time but also influence execution scores and injury risk [10].

Explosive strength refers to the ability to develop maximal force in the shortest possible time, whereas reactive strength reflects the capacity to efficiently utilize elastic energy through the stretch-shortening cycle (SSC) [11]. The SSC allows muscles and tendons to store and release elastic energy during eccentric–concentric actions. In explosive strength tasks such as the Countermovement Jump (CMJ), the SSC duration typically exceeds 350 ms, emphasizing maximal force production and rate of force development (RFD). In contrast, reactive strength—assessed through Drop Jumps (DJ)—involves much shorter SSC durations (<180 ms), testing the athlete’s ability to produce force rapidly with minimal ground contact. Both forms of strength are essential for the efficient translation of muscular power into gymnastics-specific movements [2,3,12]. Moreover, explosive strength is closely linked to maximal strength, as improvements in maximal or eccentric strength capacity provide—at least partially—the foundation for enhanced RFD and lower extremity peak power [13,14].

The development of explosive and reactive strength is strongly age- and sex-dependent [7,15,16] and requires periodized and targeted strength and power training. Research demonstrates that programs integrating maximal strength training, eccentric overload exercises, and/or plyometric drills are most effective for improving both CMJ peak power and RSI performance [15,17]. During puberty, male athletes typically experience rapid gains in muscle mass and force production due to increased androgenic hormones, leading to marked improvements in RFD and SSC efficiency [18]. In contrast, female athletes often reach a plateau earlier—around 15–16 years of age—and require more individualized training strategies to sustain progression [7]. These differences highlight the necessity for differentiated training strategies, emphasizing individualized periodization, targeted strength blocks, and plyometric progressions to counteract stagnation and optimize long-term athletic development [19,20,21,22].

To systematically monitor these physical qualities, gymnastics programs increasingly employ standardized diagnostic tests such as the Countermovement Jump (CMJ), Squat Jump (SJ) and Drop Jump (DJ). Although early performance in junior athletes shows only limited and inconsistent predictive value for later elite success [23], regular monitoring of physical capacities remains important.

These diagnostic tools are widely validated for youth and elite athletes and serve as sensitive indicators of neuromuscular performance [7,9,24]. They enable individualized feedback, facilitate monitoring of training adaptations, support early identification of athletic potential in talent pathways [25], and contribute to long-term athlete health.

The aim of this study is to establish a foundation for structured monitoring by analyzing existing performance diagnostic data from the past ten years of elite and junior athletes in Switzerland, to identify unfavorable developments within individual long-term athlete development pathways, and to provide even more precise and differentiated training recommendations. Based on this background, the present study aims, first, to analyze the performance level and longitudinal development of explosive and reactive strength of the lower extremities in junior and elite gymnasts (U14, U16, U18, and elite) using diagnostic data collected over the past ten years. Second, it aims to examine gender-specific differences in the development of these physical qualities by identifying patterns in male and female lower extremity power progression. Third, the study seeks to determine whether high levels of explosive and reactive strength in junior categories are predictive of later selection to the Swiss national elite team.

## 2. Materials and Methods

This study includes standardized performance diagnostic data on explosive and reactive strength measurements collected from Swiss elite and junior gymnasts over the past ten years. During this period, measurements were conducted on a total of 125 female gymnasts aged 7 to 26 years (mean age: 14.9 ± 4.0 years) and 109 male gymnasts aged 10 to 30 years (mean age: 18.4 ± 4.5 years). Junior athletes train between 12 and 25 h per week at different regional performance centers across Switzerland, with training volume increasing with age, while most have been engaged in elite-level gymnastics since early childhood (ages 4–6). Although all athletes follow the national development and competition program, substantial differences exist among regional performance centers in the design, implementation, and delivery of training programs, particularly with respect to strength training. Elite athletes train 25 to 30 h per week at the national performance center and follow individualized training programs targeting both technical skills and physical capacities.

The study was conducted in accordance with the Declaration of Helsinki and approved by the Cantonal Ethics Committee Bern (KEK; Project-ID: 2018-00742, 6 July 2018). Informed consent was obtained from all subjects involved in the study. All tests were conducted according to the guidelines of Maier, Gross, Trösch, Steiner, Müller, Bourban, Schärer, Hübner, Wehrlin and Tschopp [24].

### 2.1. Explosive and Reactive Strength Testing Procedures

Explosive and reactive strength were assessed using standardized testing procedures to ensure high reliability and validity [24,26]. Measurements were conducted 1–3 times per year, with most athletes measured at different time points throughout the season, providing multiple cross-sectional data points for performance analysis.

Before each test, athletes completed a checklist regarding their condition (e.g., health status, training readiness, motivation). This was followed by an individualized warm-up lasting approximately 10–15 min. Subsequently, jumps for explosive strength assessment were performed first, followed by reactive strength measurements, all conducted under the supervision of an experienced tester.

#### 2.1.1. Assessment of Explosive Strength

**Figure 1:**
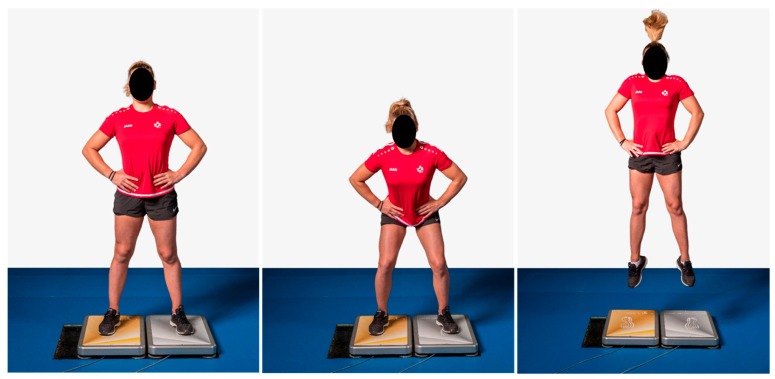
Explosive strength measurements (relative peak power) on a force plate using a countermovement jump.

Three countermovement jumps (CMJ), three squat jumps (SJ), and three single-leg CMJ (SL_CMJ) for each leg (left and right) were performed on a force plate (SPSport, Innsbruck, Austria). Explosive strength was assessed using vertical relative peak power (Pmax_rel, W/kg). A rest period of at least 5–10 s was provided between jumps to minimize fatigue. For each jump type, the average of the three best jumps was used for further analysis (Figure 1).

#### 2.1.2. Assessment of Reactive Strength

**Figure 2:**
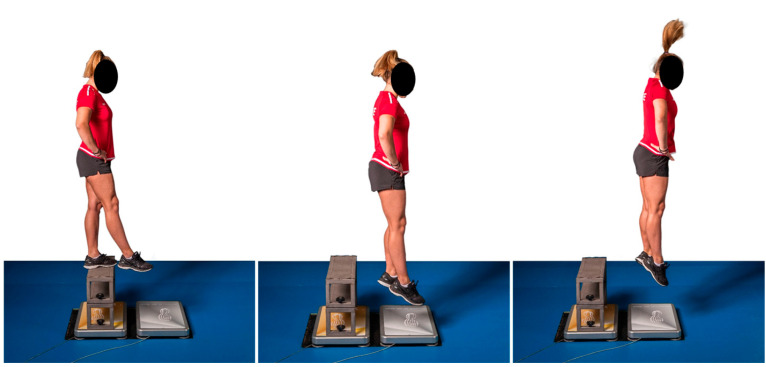
Assessment of reactive strength using a Drop Jump (40 cm drop height shown).

To assess reactive strength, drop jumps (DJ) from heights of 20, 40, and 60 cm were performed (Figure 2). For each drop height, multiple attempts (up to five jumps) were conducted until the maximum jump height with minimal ground contact time was achieved. A rest period of at least 5 s was provided between jumps. Only the maximal values were included in the analysis. For U14 athletes, the 60 cm drop height was only attempted if a clear increase in RSI2 was observed between the 20 cm and 40 cm conditions; otherwise, the 40 cm value was considered the maximal performance, reflecting the athletes’ power limitations at higher drop heights.

Based on the force plate data, the Reactive Strength Index 1 (RSI 1: jump height/ground contact time) [27] and Reactive Strength Index 2 (RSI 2: eccentric power × concentric power) were calculated using the measurement software (Cyccess Version 2.3.11, SP Sportdiagnosegeräte GmbH, Trins, Austria). Both indices reflect how efficiently athletes are able to rebound with minimal ground contact time and minimal knee flexion (Figure 2). RSI2 reflects the athlete’s ability to absorb kinetic energy during ground contact and immediately reinvest it, capturing both the eccentric (braking) and concentric (propulsive) phases of the stretch-shortening cycle. This index is particularly relevant in gymnastics, where high ground reaction forces and body weight significantly influence performance. RSI1 reflects jump height relative to contact time and is more sensitive in movements with lower braking demands. Together, these indices provide complementary information on deceleration capacity and re-acceleration quality [24].

### 2.2. Data Analysis

For the analysis, the data were stratified into several groups based on age category (U14, U16, U18, and Elite), gender (female/male), and national team affiliation (current or former elite national team members vs. no national team affiliation). Statistical analyses were not performed for groups with small sample sizes (*n* < 10) to ensure the interpretability of the results.

The data were first tested for normality. Means and standard deviations were calculated for each of the above-mentioned groups. Group differences were then examined using one-way analysis of variance (ANOVA) and post hoc *t*-tests. Homogeneity of variances was assessed using Levene’s test. Effect sizes were calculated for all statistical tests. For ANOVA, eta squared (*η*^2^) was used, whereas Cohen’s *d* was applied for *t*-tests. For ANOVA, effect sizes were interpreted as small (*η*^2^ < 0.01), medium (0.01 ≤ *η*^2^ < 0.06), and large (*η*^2^ ≥ 0.06). For *t*-tests, effect sizes were classified as small (*d* < 0.2), medium (0.2 ≤ *d* < 0.5), and large (*d* ≥ 0.5).

To visually compare the data between male and female athletes, in addition to comparing different age categories using *t*-tests, the data were sorted by the athletes’ age. Consecutive groups of five tests were aggregated, and a moving average was calculated. The same procedure was applied to the standard deviation of the corresponding five datasets. The resulting data were then fitted using a third- or fourth-order polynomial, and the coefficient of determination (*R*^2^) was calculated. The significance level was set at *p* < 0.05. All statistical analyses were performed using JASP (Version 0.17.1.0, University of Amsterdam, Amsterdam, The Netherlands).

## 3. Results

Four datasets were excluded from the analyses because the tests were conducted under injury-related limitations, as documented in the measurement software. In addition, reactive strength values for male U14 athletes were excluded due to an insufficient sample size (*n* < 10). Raw data can be found in the Supplementary Materials (Table S1).

### 3.1. Development of Explosive and Reactive Strength

The development of explosive and reactive strength showed significant age-related increases in both male and female athletes. Analysis of variance revealed a significant increase in values across the age categories (U14, U16, U18, and Elite) for both genders (*p* < 0.001). The estimated effect sizes (*η*^2^) exceeded 0.14 for all analyses, indicating large effects according to Cohen [28].

The differences between the U14 and U16 categories were particularly pronounced in female athletes. Across all explosive strength tests, U16 athletes demonstrated significantly higher values than those in the U14 category (CMJ: +12.93%, *p* < 0.001, *d* = 1.10; SL_CMJ: +12.65%, *p* < 0.001, *d* = 1.08; SJ: +8.74%, *p* < 0.001, *d* = 0.80). Reactive strength was also significantly higher in the U16 category across all tests (RSI 1: +15.55%, *p* < 0.001, *d* = 0.84; RSI 2: +21.18%, *p* < 0.001, *d* = 0.61).

In contrast, no significant differences were observed between the U16 and U18 categories in any test (*p* > 0.05). This applied to both explosive strength (CMJ: −0.53%, *d* = 0.06; SL_CMJ: +0.79%, *d* = 0.08; SJ: −0.98%, *d* = 0.10) and reactive strength (RSI 1: +2.67%, *d* = 0.21; RSI 2: +9.19%, *d* = 0.34).

Between the U18 and Elite categories, moderate but significant improvements were observed in explosive strength for SL_CMJ (+5.27%, *d* = 0.52) and SJ (+5.98%, *d* = 0.52), whereas no significant difference was found for CMJ (+3.78%, *d* = 0.39; *p* > 0.05). Regarding reactive strength, a significant increase was observed only for RSI 2 (+16.00%, *d* = 0.62), while RSI 1 showed no significant difference (+5.90%, *d* = 0.42; *p* > 0.05) (Table 1).

In male athletes, explosive strength increased significantly between the U14 and U16 categories (CMJ bilateral: +14.94%, *p* < 0.001, *d* = 1.06; CMJ unilateral: +13.66%, *p* < 0.001, *d* = 1.16; SJ: +15.72%, *p* < 0.001, *d* = 1.25). Reactive strength also showed significant increases over this period (RI 1: +15.55%, *p* < 0.001, *d* = 0.79; RI 2: +21.18%, *p* < 0.05, *d* = 0.52).

This trend continued between the U16 and U18 categories, with further significant improvements observed in both explosive strength (CMJ bilateral: +9.34%, *p* < 0.001, *d* = 0.81; CMJ unilateral: +10.68%, *p* < 0.001, *d* = 1.12; SJ: +11.13%, *p* < 0.001, *d* = 1.06) and reactive strength (RI 1: +16.98%, *p* < 0.001, *d* = 1.11; RI 2: +24.62%, *p* < 0.001, *d* = 0.90).

Between the U18 and Elite categories, elite athletes again demonstrated moderately but significantly higher values in CMJ bilateral (+5.64%, *p* < 0.01, *d* = 0.51) and CMJ unilateral (+6.63%, *p* < 0.01, *d* = 0.59), whereas no significant difference was observed for SJ (+2.34%, *p* > 0.05, *d* = 0.25). Similarly, no significant increases were found in reactive strength between the U18 and Elite categories (RI 1: +4.09%, *p* > 0.05, *d* = 0.35; RI 2: +4.69%, *p* > 0.05, *d* = 0.22) (Table 1).

### 3.2. Gender-Specific Differences in the Development of Explosive and Reactive Strength

The sex-specific analysis revealed no significant differences in explosive strength between male and female athletes in the U14 category (CMJ: M > F: +3.28%; SL_CMJ: M > F: +0.37%; SJ: F > M: +0.16%; *p* > 0.05; *d* < 0.5). In the U16 category, male athletes demonstrated significantly higher explosive strength values than females in the CMJ (+5.12%, *p* < 0.01, *d* = 0.463) and SJ (+6.25%, *p* < 0.001, *d* = 0.577), whereas no significant sex difference was observed in the SL_CMJ (+1.30%, *p* > 0.05, *d* = 0.138). From the U18 category onwards, males exhibited significantly greater explosive strength across all tests (CMJ: +15.55%, *d* = 1.458; SL_CMJ: +11.27%, *d* = 1.175; SJ: +19.23%, *d* = 1.916; all *p* < 0.001). This trend persisted at the elite level, where males outperformed females in all explosive strength measures (CMJ: +17.62%, *d* = 1.486; SL_CMJ: +12.67%, *d* = 1.062; SJ: +15.13%, *d* = 1.350; all *p* < 0.001).

With respect to reactive strength, no significant sex differences in RSI 1 or RSI 2 were observed in the U14 and U16 categories (*p* > 0.05; *d* ≤ 0.34). However, in the U16 category, female athletes achieved significantly higher RSI 2 values than males (+16.47%, *p* < 0.001, *d* = 0.692), while no significant difference was found for RSI 1 (+1.38%, *p* > 0.05, *d* = 0.096). From the U18 category onwards, significant sex-related differences in reactive strength became evident. Male athletes demonstrated significantly higher RSI 1 values in both the U18 (+12.36%, *p* < 0.001, *d* = 0.924) and elite (+10.44%, *p* < 0.001, *d* = 0.81) categories. In contrast, female athletes exhibited significantly higher RSI 2 values in the U16 (+16.47%, *p* < 0.001, *d* = 0.69) and elite (+13.96%, *p* < 0.001, *d* = 0.76) categories, whereas no significant sex difference was observed for RSI 2 in the U18 category (−4.66%, *p* > 0.05, *d* = 0.19).

**Figure 3:**
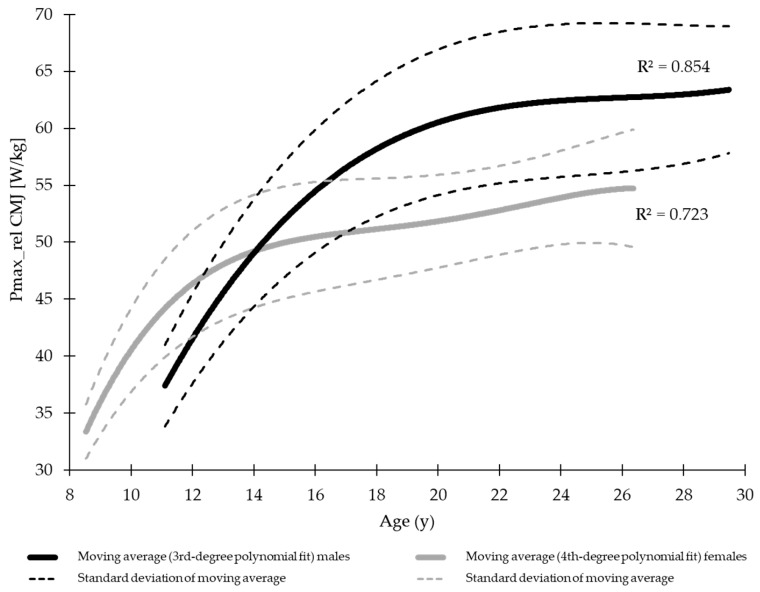
Polynomially fitted moving average (±SD) of explosive strength (relative peak power during the countermovement jump, Pmax_rel CMJ) in male and female gymnasts (R^2^: proportion of variance explained by the polynomial fit).

**Figure 4:**
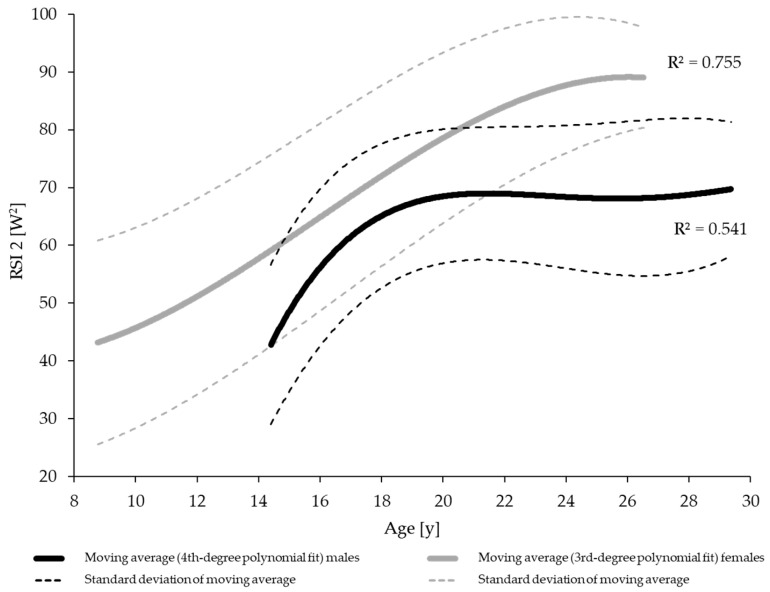
Polynomially fitted moving average (±SD) of reactive strength (Reactive Strength Index 2 (RSI 2: eccentric power × concentric power) in male and female gymnasts (R^2^: proportion of variance explained by the polynomial fit).

A visual comparison of the mean development of explosive and reactive strength across age is presented in Figure 3 and Figure 4.

### 3.3. Performance Differences Between Later Elite National Team Athletes and Non-Elite National Team Athletes

The only parameter in explosive strength showing a tendency toward better performance in female athletes who later became members of the national team compared with athletes who were never part of the national team was observed in the U14 category for CMJ (national squad: 47.35 ± 5.14 W·kg^−1^; non-national team: 44.97 ± 5.45 W·kg^−1^; *p* = 0.08; *d* = 0.45). All other explosive strength parameters (SJ, SL-CMJ) were small and non-significant across age categories, with percentage differences ranging from +0.77% to +5.29% and effect sizes from *d* = 0.08 to 0.32. In male athletes, differences ranged from −6.43% to +8.02% (*d* = 0.25–0.75), with isolated significant differences observed in U16 for CMJ (non-national team: 55.06 ± 6.54 W·kg^−1^; national team: 51.52 ± 5.65 W·kg^−1^; *p* = 0.031; *d* = 0.57) and U18 for SL_CMJ (national team: 36.24 ± 2.89 W·kg^−1^; non-national team: 34.68 ± 2.34 W·kg^−1^; *p* = 0.043; *d* = 0.74).

**Figure 5:**
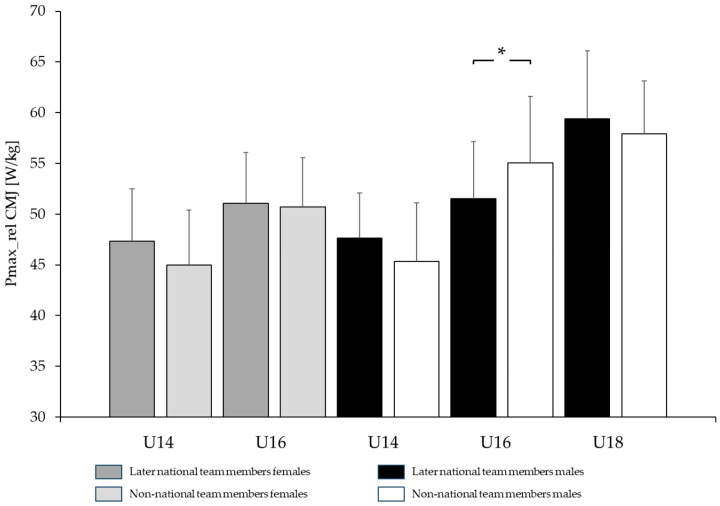
Comparison of explosive strength values (peak power [Pmax_rel] during countermovement jumps) between men and women across different age categories, comparing athletes who later became members of the national team with those who never qualified for the national team (*: *p* < 0.05).

**Figure 6:**
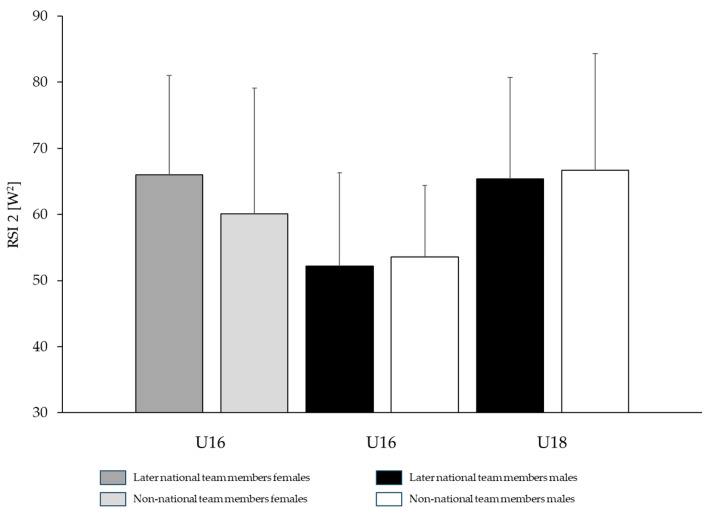
Comparison of reactive strength values (Reactive Strength Index 2 from drop jumps) between men and women across different age categories, comparing athletes who later became members of the national team with those who never qualified for the national team.

In female athletes, differences in reactive strength (RSI 1 and RSI 2) were trivial to small and non-significant across all age categories (national team > non-national team: +1.10% to +9.89%, *d* = 0.00–0.09). In contrast, male athletes exhibited differences ranging from −8.86% to −2.02% (non-national team > national team; *d* = 0.08–0.57), which likewise remained non-significant across all age categories. All comparisons, including CMJ, SJ, and reactive index measures across U14–U18 males and females, exhibited low to moderate power due to small sample sizes. For illustrative comparisons of CMJ and RSI 2 between athletes who later reached the national team and those who did not, Figure 5 and Figure 6 are provided.

## 4. Discussion

This article examined the development of explosive and reactive strength in Swiss artistic gymnasts based on standardized performance diagnostic tests collected over the past ten years, covering age categories from U14 to elite male and female athletes. Furthermore, differences between age categories and sex-specific developmental patterns were analyzed, as well as differences between athletes who were later selected for the elite national squad and those who were not.

The main findings indicate that male athletes show a continuous increase in explosive strength across age categories up to the elite level, whereas female gymnasts exhibit a stagnation in explosive strength after the age of 16. In contrast, female athletes demonstrate continuous development in reactive strength (RSI 2) across all age categories, while male athletes stagnate in reactive strength from the elite level onwards. Comparisons between male and female athletes within the same age categories revealed similar levels of explosive strength up to the age of 16; however, female athletes displayed superior reactive strength from the U16 category onwards. Interestingly, only minor differences in explosive and reactive strength were observed between athletes who were later selected for the national team and those who were never selected.

### 4.1. Development of Explosive and Reactive Strength

Explosive and reactive strength increased significantly with age in both female and male athletes, with large overall effects observed across all age categories (U14 to Elite). In female athletes, the most pronounced improvements occurred between U14 and U16 across all measures of explosive and reactive strength (*p* < 0.001; +12.93%). This finding is consistent with Tingelstad et al. [29], who also reported the greatest improvements in jump performance among elite female athletes between 14 and 15 years of age (+7.2%). In contrast to their results, which showed a small but continuous increase in jump performance after the age of 16 (approximately 5% per year), CMJ performance in the present study largely stagnated between the U16 and U18 categories.

From U18 to Elite, only selective and moderate improvements (*p* < 0.05) were observed in females, mainly in explosive strength (SL_CMJ) and one reactive strength parameter (RSI 2). This reflects the sport-specific importance of one-legged jump-offs in gymnastics, as many gymnastic jumps are performed unilaterally [7] and reactive strength seems to be one of the most important physical preconditions in order to reach a sufficient height to perform the most difficult gymnastics elements on the floor, vault and balance beam [3,30,31]. Conversely, it may be assumed that improvements in explosive strength were primarily driven by sport-specific training and gymnastics-specific skills. In this context, general physical capacities—such as maximal strength, which is considered a fundamental prerequisite for the development of explosive strength and muscular power of the lower extremities [32]—may not be sufficiently emphasized in training. At the same time, an increasing focus on the acquisition and refinement of gymnastics-specific skills may limit the training volume devoted to general strength and power development. As a result, the athletes’ neuromuscular potential might not be fully exploited, thereby constraining further improvements in explosive performance.

In male athletes, explosive and reactive strength increased consistently from U14 to U18, with significant improvements observed at each age transition (*p* < 0.001), albeit with progressively smaller gains across successive age groups. The largest performance increases typically occur during the period of peak height velocity (PHV) and the early post-pubertal phase, when rapid biological maturation and hormonal changes strongly enhance strength and power development [33]. Consequently, the magnitude of improvements gradually declines thereafter. From U18 to the elite level, performance gains were more limited and primarily confined to CMJ and SL_CMJ performance (*p* < 0.01). A gradual reduction in improvements in jump performance and other power-related measures has also been reported by several authors [29,33,34,35] and may reflect both the diminishing effects of maturation-related adaptations and a decreasing effectiveness of training as athletes approach their performance ceiling.

### 4.2. Gender-Specific Differences in the Development of Explosive and Reactive Strength

The results indicate that gender-related differences in explosive strength increase with age. In the U14 category, no significant gender differences were observed (*p* > 0.05), with only small effect sizes across all tests. From the U16 category onward, clear differences emerged, with male athletes achieving approximately 5.1% higher CMJ performance (*p* = 0.007) and 6.3% higher SJ performance (*p* < 0.001) compared to female athletes. These differences became substantially more pronounced in the U18 category, where males outperformed females by about 15.6% in CMJ, 11.3% in SL_CMJ, and 19.2% in SJ (all *p* < 0.001). Similar gender-related differences were also evident at the elite level, with male athletes achieving approximately 17.6% higher CMJ, 12.7% higher SL_CMJ, and 15.1% higher SJ performance compared to female athletes (all *p* < 0.001).

This finding is partly in line with the well-documented age-related development of explosive strength assessed by the countermovement jump (CMJ), whereby sex-related differences increase progressively with advancing age [36]. However, the authors of the referenced study reported that in children and adolescents not involved in elite sports, sex-related differences already begin to widen from approximately 12 years of age. In that cohort, sex differences reached +9.5% in 11–12-year-olds and +11.0% in 13–14-year-olds, with the most pronounced divergence occurring during and shortly after puberty (+32.5% in 15–16-year-olds). Thereafter, sex-related differences increased only marginally, reaching a plateau of +37.4% in 23–24-year-olds.

Interestingly, in contrast to these findings, sex differences in elite youth gymnasts appear to emerge two years later, with statistically significant differences observed only from approximately 15–16 years of age (U16) in the present study. Furthermore, sex-related differences in our cohort are similar to those reported by the normative data by Hübner, Fischer, Lüthy and Tschopp [7]. These observations suggest that systematic training and long-term athletic development may exert a moderating effect on the magnitude of sex-related differences in explosive strength, particularly during early and mid-adolescence, potentially delaying the manifestation of pronounced performance disparities between male and female athletes. Our findings are supported by Martin et al. [37], who observed no significant sex-related differences up to the age of 14 years (*p* > 0.05), whereas clear performance advantages in favor of boys emerged thereafter. These differences were attributed to qualitative neuromuscular factors, including a higher proportion of type II muscle fibers, greater glycolytic capacity, and superior motor coordination, which become increasingly relevant during and after puberty.

Reactive strength showed no relevant sex-related differences in the U14 category and only minor differences in U16 athletes. Notably, female athletes in the U16 category demonstrated higher RSI 2 values than males (+16.5%; *p* < 0.001), while RSI 1 did not differ meaningfully between sexes (+1.4%; *p* > 0.05). From the U18 category onwards, sex-specific patterns became more pronounced. Male athletes exhibited superior RSI 1 values in both U18 (+12.4%; *p* < 0.001) and elite athletes (+10.4%; *p* < 0.001). In contrast, females outperformed males in RSI 2 at the elite level (+14.0%; *p* < 0.001), whereas no meaningful sex difference in RSI 2 was observed in the U18 category (−4.7%; *p* > 0.05).

RSI 1 is calculated as the ratio of jump height to ground contact time. Within this parameter, the duration of the concentric acceleration phase and the athlete’s rate of force development (RFD) are critical determinants of performance. Physiologically, male athletes generally exhibit a higher proportion of type II muscle fibers, greater maximal voluntary force, and enhanced neuromuscular activation. Together, these factors make males more likely to achieve superior concentric force production and acceleration capabilities, enabling them to achieve greater takeoff velocities despite comparable ground contact times. Compared to the study by Zemková and Štefániková [36], which examined the development of reactive strength (RSI 1) in non-elite athletes, where males consistently outperformed females across all age groups and the sex-related differences increased with age (e.g., 10% at 7 years and 31% at 24 years), it becomes evident that gymnastics-specific training substantially reduces these differences. Similar to what has been observed for explosive strength, professional training appears to enable female athletes to utilize a much greater proportion of their neuromuscular potential than is typically observed in males. This suggests that structured, sport-specific training can mitigate the typical sex-related performance gap and enhance the functional expression of reactive strength in female elite athletes.

In contrast, RSI 2 represents the ability to store and reutilize elastic energy via the stretch-shortening cycle (SSC). In the present study, female athletes in certain age categories exhibited higher RSI 2 values than their male counterparts, indicating a potentially greater efficiency in converting eccentric braking forces into elastic recoil under these conditions. This finding suggests that reactive strength plays a particularly important role in women’s gymnastics. This interpretation is supported by the continuous development of RSI 2 observed in female athletes and by the fact that three of the four women’s apparatuses rely predominantly on lower-limb actions.

Conversely, in men’s gymnastics, only two apparatuses place a decisive emphasis on lower-limb physical capacities. In addition, the increased elastic properties of modern gymnastics apparatuses require take-offs to be performed with increasingly extended lower limbs, a movement pattern closely associated with reactive strength capabilities. While male gymnasts may benefit from these enhanced elastic properties due to their generally higher body mass and thus greater ability to exploit the spring effect of the apparatus, female gymnasts likely need to compensate through superior reactive strength capacities. This may explain why higher RSI 2 values appear particularly relevant for female gymnasts in achieving elements of comparable technical difficulty. Overall, these findings suggest that reactive strength development follows sex- and load-specific trajectories, with training and long-term athletic development influencing how braking performance, ground contact time, and elastic energy utilization interact across maturation. Furthermore, this study supports the conclusions of Lehnert et al. [38], indicating that SSC-specific training should be incorporated into training programs at an early age, even in children and prepubescent adolescents, where it has been shown to produce substantial improvements in reactive strength.

### 4.3. Performance Differences Between Later Elite National Team Athletes and Non-Elite National Team Athletes

Interestingly, the comparison between male and female gymnasts who are or have been part of the elite national team and those who have never been selected revealed no statistically significant differences (*p* > 0.05) in most performance measures. This suggests that selection for the elite national team may be primarily based on technical proficiency, while physical strength attributes such as explosive and reactive strength receive comparatively less emphasis. Notably, the largest relative differences were observed in U14 gymnasts for explosive strength, with national team athletes outperforming non-national team athletes by approximately 8–9% in both males and females, and in U16 female gymnasts for reactive strength (+10%). In other age categories, performance values were either comparable or, in some cases, non-national team athletes even outperformed their national team counterparts.

Although these differences were not statistically significant, they indicate that physical prerequisites may still play a relevant role, particularly in the selection process, with a tendency for athletes possessing superior physical capacities to be favored, especially among females. This observation aligns with the findings of one of our previous studies [3], which highlighted the influence of physical prerequisites on jump height across different apparatuses.

Nevertheless, the results underscore the central role of technical skill in gymnastics. Physical capabilities form the essential foundation for the successful execution of technically demanding elements, which are critical for competitive success. For coaches, this implies that while technical mastery is paramount, physical conditioning should be developed in parallel. In female athletes, this is particularly important, as performances on three of the four apparatuses heavily rely on lower-extremity strength. In male athletes, physical conditioning should also be developed to a functional level, as suggested in one of our previous studies [39], where it contributes meaningfully to performance without overshadowing technical execution. Ultimately, prioritizing technique in conjunction with targeted physical development is key to achieving long-term athletic success.

### 4.4. Limitations

Biological maturation may confound the interpretation of strength and power development in youth gymnastics, as athletes were grouped by chronological age rather than maturation status. Around Peak Height Velocity (PHV), substantial inter-individual differences in neuromuscular development occur. Artistic gymnastics also favors later-maturing athletes, whose smaller body size and lower body mass provide performance advantages [40]. Consequently, gymnasts often mature later than age-matched peers, which may partly explain variability within age groups and the apparent performance plateau observed in older female athletes. Future studies should include maturation indicators to better separate training effects from growth-related adaptations.

A further limitation of this study is the small sample size in several age and sex subgroups, which reduced statistical power for comparisons between athletes who later reached the national team and those who did not. Therefore, further investigation of this comparison is warranted.

## 5. Conclusions

This study demonstrates clear age- and gender-specific differences in explosive and reactive strength development among gymnasts. Male athletes show marked improvements in explosive strength during puberty, whereas female athletes tend to plateau in explosive strength around the age of 16 years. At the same time, reactive strength appears to gain increasing importance, particularly in women’s artistic gymnastics, due to the improved elastic properties of modern apparatuses and the rising difficulty demands of competitive routines. Although technical skill and coordination remain the primary determinants of elite selection, physical prerequisites provide a crucial foundation for performance, injury prevention, and long-term athlete development. The graphical representations of strength development presented in this study offer coaches and researchers a practical tool to monitor physical capacities, detect potential negative trends at an early stage, and identify periods of accelerated development that may be particularly suitable for targeted technical interventions. Accordingly, the implementation of age- and gender-specific, progressively structured strength training in parallel with technical development may enhance performance, robustness, and career longevity. Ultimately, the systematic integration of physical and technical preparation is essential for the sustainable advancement of artistic gymnastics.

## Figures and Tables

**Table 1:** Means, standard deviations, and significant differences compared with previous age categories for explosive strength (relative peak power [Pmax_rel]) measured during single-leg (SL) and double-leg countermovement jumps (CMJ) as well as single-leg squat jumps (SJ), and reactive strength during drop jumps (Reactive Strength Index 1 [RSI1]: contact time/jump height; Reactive Strength Index 2 [RSI2]: eccentric power × concentric power), assessed using a force plate. Values marked with asterisks indicate significant differences compared with the previous age category: * *p* < 0.05, ** *p* < 0.01, *** *p* < 0.001.

Age		Body Mass [kg]	CMJ [W/kg]		SL_CMJ [W/kg]		SJ [W/kg]		RSI 1 [h_s_/10·t_k_]		RSI 2 [W^2^]	
Women	U14	35.49 ± 6.15	*n* = 190	45.18 ± 5.45	*n* = 184	28.07 ± 3.49	*n* = 188	42.68 ± 4.79	*n* = 51	18.14 ± 3.97	*n* = 51	52.36 ± 19.20
	U16	52.99 ± 4.53	*n* = 78	51.02 ± 4.95 ***	*n* = 66	31.62 ± 2.67 ***	*n* = 67	46.41 ± 4.30 ***	*n* = 53	20.96 ± 2.60 ***	*n* = 53	63.45 ± 16.97 *
	U18	57.29 ± 5.13	*n* = 64	50.75 ± 4.90	*n* = 60	31.87 ± 3.29	*n* = 60	45.96 ± 4.65	*n* = 40	21.52 ± 2.86	*n* = 40	69.28 ± 17.64
	Elite	57.33 ± 2.41	*n* = 71	52.67 ± 4.96	*n* = 65	33.55 ± 3.23 *	*n* = 67	48.71 ± 5.78 *	*n* = 44	22.79 ± 3.21	*n* = 44	80.37 ± 17.99 *
Men	U14	38.30 ± 5.40	*n* = 80	46.66 ± 6.71	*n* = 79	28.18 ± 3.34	*n* = 80	42.61 ± 5.08	*n* = 7	17.92 ± 4.09	*n* = 4	59.19 ± 9.30
	U16	50.96 ± 7.71	*n* = 62	53.63 ± 6.39 ***	*n* = 62	32.03 ± 3.28 ***	*n* = 63	49.31 ± 5.66 ***	*n* = 49	20.67 ± 3.40	*n* = 40	53.00 ± 12.14
	U18	60.91 ± 6.89	*n* = 53	58.64 ± 5.98 ***	*n* = 49	35.45 ± 2.72 ***	*n* = 53	54.80 ± 4.58 ***	*n* = 46	24.18 ± 2.90 ***	*n* = 42	66.05 ± 16.33 ***
	Elite	66.62 ± 4.88	*n* = 169	61.95 ± 6.71 **	*n* = 133	37.80 ± 4.33 **	*n* = 171	56.08 ± 5.33	*n* = 135	25.17 ± 2.86	*n* = 111	69.15 ± 13.29

## Data Availability

The original contributions presented in this study are included in the article/Supplementary Materials. Further inquiries can be directed to the corresponding author.

## Supplementary Materials

The following supporting information can be downloaded at: https://www.mdpi.com/article/10.3390/jfmk11010096/s1, Table S1: Raw data of explosive (CMJ, SJ, SL_CMJ) and reactive strength (RSI 1, RSI 2) measurements (*: Athletes of national teams).

## Abbreviations

The following abbreviations are used in this manuscript:

U	Under (Age Category)
SSC	Stretch-Shortening Cycle
CMJ	Countermovement Jump
SJ	Squat Jump
SL_CMJ	Single-Leg Countermovement Jump
DJ	Drop Jump
RFD	Rate of Force Development
Pmax_rel	Relative Peak Power
RSI 1	Reactive Strength Index 1
RSI 2	Reactive Strength Index 2
SD	Standard Deviation

## References

[1] Heinen T., Naundorf F., Scharenberg S., Schlegel K., Krug J., Güllich A., Krüger M. (2019). Gerät- und Kunstturnen. Grundlagen von Sport und Sportwissenschaft: Handbuch Sport und Sportwissenschaft.

[2] Schärer C., Haller N., Taube W., Hübner K. (2019). Physical determinants of vault performance and their age-related differences across male junior and elite top-level gymnasts. PLoS ONE.

[3] Schärer C., Reinhart L., Hübner K. (2023). Age-related differences between maximum flight height of basic skills on floor, beam and vault and physical condition of youth female artistic gymnasts. Sports.

[4] Bradshaw E.J., Le Rossignol P., Williams M., Lorenzen C. Novel insights on lower limb musculoskeletal health and performance in pre-adolescent and adolescent gymnasts. Proceedings of the 24th International Symposium on Biomechanics in Sports.

[5] Aleksić-Veljković A., Madić D., Vukadinović M., Herodek K., Marković K.Ž., Badić A. (2013). Jumping Abilities in Young Female Gymnasts: Age-Group Differences. Exerc. Qual. Life.

[6] Mcneal J.R., Sands W.A., Shultz B.B. (2007). Muscle activation characteristics of tumbling take-offs. Sports Biomech..

[7] Hübner K., Fischer K., Lüthy F., Tschopp M. (2013). Explosivkraftniveau der unteren Extremitäten bei Schweizer Nachwuchsathleten. Schweiz. Z. Sportmed. Sporttraumatol..

[8] Dallas G.C., Kirialanis P., Dallas C.G., Mellos V. (2017). The effect of training in maximal isometric strength in young artistic gymnasts. Sci. Gymnast. J..

[9] French D.N., Gómez A.L., Volek J.S., Rubin M.R., Ratamess N.A., Sharman M.J., Gotshalk L.A., Sebastianelli W.J., Putukian M., Newton R.U. (2004). Longitudinal tracking of muscular power changes of NCAA Division I collegiate women gymnasts. J. Strength Cond. Res..

[10] Bradshaw E.J., Hume P.A. (2012). Biomechanical approaches to identify and quantify injury mechanisms and risk factors in women’s artistic gymnastics. Sports Biomech..

[11] Maffiuletti N.A., Aagaard P., Blazevich A.J., Folland J., Tillin N., Duchateau J. (2016). Rate of force development: Physiological and methodological considerations. Eur. J. Appl. Physiol..

[12] Bradshaw E.J., Rossignol P.L. (2004). Gymnastics: Anthropometric and biomechanical field measures of floor and vault ability in 8 to 14 year old talent-selected Gymnasts. Sports Biomech..

[13] Beattie K., Carson B.P., Lyons M., Kenny I.C. (2017). The relationship between maximal strength and reactive strength. Int. J. Sports Physiol. Perform..

[14] Smilios I., Sotiropoulos K., Christou M., Douda H., Spaias A., Tokmakidis S.P. (2013). Maximum power training load determination and its effects on load-power relationship, maximum strength, and vertical jump performance. J. Strength Cond. Res..

[15] Moeskops S., Oliver J.L., Read P.J., Cronin J.B., Myer G.D., Lloyd R.S. (2019). The physiological demands of youth artistic gymnastics: Applications to strength and conditioning. Strength Cond. J..

[16] Moeskops S. (2020). The Effects of Growth, Maturation and Training on Strength and Power Development in Young Artistic Female Gymnasts. Doctoral Dissertation.

[17] Moeskops S., Oliver J., Read P.J., Haff G.G., Myer G.D., Lloyd R.S. (2022). Effects of a 10-Month Neuromuscular Training Program on Strength, Power, Speed and Vault Performance in Young Female Gymnasts.

[18] Lesinski M., Schmelcher A., Herz M., Puta C., Gabriel H., Arampatzis A., Laube G., Büsch D., Granacher U. (2020). Maturation-, age-, and sex-specific anthropometric and physical fitness percentiles of German elite young athletes. PLoS ONE.

[19] Granacher U., Lesinski M., Büsch D., Muehlbauer T., Prieske O., Puta C., Gollhofer A., Behm D.G. (2016). Effects of resistance training in youth athletes on muscular fitness and athletic performance: A conceptual model for long-term athlete development. Front. Physiol..

[20] Lloyd R.S., Cronin J.B., Faigenbaum A.D., Haff G.G., Howard R., Kraemer W.J., Micheli L.J., Myer G.D., Oliver J.L. (2016). National Strength and Conditioning Association position statement on long-term athletic development. J. Strength Cond. Res..

[21] Faigenbaum A.D., Lloyd R.S., MacDonald J., Myer G.D. (2016). Citius, Altius, Fortius: Beneficial effects of resistance training for young athletes: Narrative review. Br. J. Sports Med..

[22] Long C., Ranellone S., Welch M. (2024). Strength and Conditioning in the Young Athlete for Long-Term Athletic Development. HSS J..

[23] Güllich A., Barth M., Macnamara B.N., Hambrick D.Z. (2023). Quantifying the extent to which successful juniors and successful seniors are two disparate populations: A systematic review and synthesis of findings. Sports Med..

[24] Maier T., Gross M., Trösch S., Steiner T., Müller B., Bourban P., Schärer C., Hübner K., Wehrlin J., Tschopp M. (2016). Manual Leistungsdiagnostik.

[25] Marina M., Jemni M. (2014). Plyometric training performance in elite-oriented prepubertal female gymnasts. J. Strength Cond. Res..

[26] Markovic G., Dizdar D., Jukic I., Cardinale M. (2004). Reliability and factorial validity of squat and countermovement jump tests. J. Strength Cond. Res..

[27] Flanagan E.P., Comyns T.M. (2008). The use of contact time and the reactive strength index to optimize fast stretch-shortening cycle training. Strength Cond. J..

[28] Cohen J. (1988). Statistical Power Analysis for the Behavioral Sciences.

[29] Tingelstad L.M., Raastad T., Till K., Luteberget L.S. (2023). The development of physical characteristics in adolescent team sport athletes: A systematic review. PLoS ONE.

[30] Dallas G., Dallas C., Pappas P., Paradisis G. (2023). Acute effect of bounce drop jump and countermovement drop jump with and without additional load on jump performance parameters and reactive strength index on young gymnasts. Hum. Mov..

[31] Feng D., Yang W., Li L. (2024). Countermovement jump and reactive strength index of artistic gymnasts improve more with cluster-based plyometric training than with traditional methods. Sci. Rep..

[32] Cronin J.B., Hansen K.T. (2005). Strength and power predictors of sports speed. J. Strength Cond. Res..

[33] Guimarães E., Maia J.A., Williams M., Sousa F., Santos E., Tavares F., Janeira M.A., Baxter-Jones A.D. (2021). Muscular strength spurts in adolescent male basketball players: The INEX study. Int. J. Environ. Res. Public Health.

[34] Mendez-Villanueva A., Buchheit M., Kuitunen S., Douglas A., Peltola E., Bourdon P. (2011). Age-related differences in acceleration, maximum running speed, and repeated-sprint performance in young soccer players. J. Sports Sci..

[35] Lloyd R.S., Oliver J.L. (2012). The youth physical development model: A new approach to long-term athletic development. Strength Cond. J..

[36] Zemková E., Štefániková G.K. (2025). Age- and gender-related differences in explosive leg muscle function with respect to jump tests used: A comparative study. BMC Sports Sci. Med. Rehabil..

[37] Martin R.J., Dore E., Twisk J., van Praagh E., Hautier C.A., Bedu M. (2004). Longitudinal changes of maximal short-term peak power in girls and boys during growth. Med. Sci. Sports Exerc..

[38] Lehnert M., Psotta R., Helešic J. (2022). Influence of chronological age on reactive strength in 8–13-year-old female figure skaters. J. Phys. Educ. Sport.

[39] Schärer C., Lehmann T., Naundorf F., Taube W., Hübner K. (2019). The faster, the better? Relationships between run-up speed, the degree of difficulty (D-score), height and length of flight on vault in artistic gymnastics. PLoS ONE.

[40] Malina R.M., Baxter-Jones A.D., Armstrong N., Beunen G.P., Caine D., Daly R.M., Lewis R.D., Rogol A.D., Russell K. (2013). Role of intensive training in the growth and maturation of artistic gymnasts. Sports Med..

