# Supplementary material for: Development of Lower-Limb Power Across Age and Sex in Junior and Elite Artistic Gymnasts
Source: J Funct Morphol Kinesiol. 2026 Feb 26;11(1):96. doi: 10.3390/jfmk11010096 (PMC13028325; doi:10.3390/jfmk11010096)
Supplement: Supplementary file 1 [file jfmk-11-00096-s001.zip › jfmk-4153952-supplementary.pdf]

Table S1: Raw data of explosive (CMJ, SJ, SL_CMJ) and reactive strength (RSI 1, RSI 2) measurements (*: Athletes of national teams).

**Athlete Nr.**	**Gender**	**Category**	**Age [y]**	**BodyMass [kg]**	**CMJ_Pmax_rel [W/kg]**	**SJ_Pmax_rel [W/kg]**	**SL_CMJ [W/kg]**	**RSI 1 [hs/10·tk]**	**RSI 2 [W²]**
18*	F	Elite	18.20	65.30	59.36	49.86	32.04	25.73	75.19
18*	F	Elite	18.46	69.30	51.29	49.12	29.51		
18*	F	Elite	18.79	67.70	56.06	49.10	32.96	26.67	84.50
18*	F	Elite	19.35	65.70	58.79				
18*	F	Elite	19.35	65.50	54.66				
24*	F	Elite	18.47	54.00	46.92	44.86	32.19		
24*	F	Elite	19.07	52.20	52.34	42.07	32.88	24.31	82.29
24*	F	Elite	19.48	56.70	42.84	38.05	28.15		
24*	F	Elite	19.54	54.80	45.52	40.16			
24*	F	Elite	20.32	57.90	48.26	42.93	32.39	20.68	57.56
24*	F	Elite	20.94	60.00	44.61	42.97	31.47	18.89	27.55
26*	F	Elite	18.23	59.60	58.42	56.47	32.92		
26*	F	Elite	18.91	59.00	53.18	54.35	32.66	28.97	99.50
26*	F	Elite	19.33	58.70	53.19	53.79			
26*	F	Elite	19.98	58.90	52.83	52.32	34.42	28.33	103.71
26*	F	Elite	20.21	58.70	54.55	53.62	31.64	28.89	110.15
26*	F	Elite	21.12	59.70	57.86	56.43			
26*	F	Elite	21.13	60.00	59.25				
34*	F	Elite	19.22	55.00	47.42	46.23	26.17	22.41	72.19
34*	F	Elite	20.59	57.10	54.43	44.84	29.01	21.99	84.37
34*	F	Elite	20.76	59.90	43.85	45.80	26.34	20.01	61.75
34*	F	Elite	21.97	57.30	50.97	42.56	30.30	18.13	44.29
34*	F	Elite	22.53	56.70	52.16	50.07	33.30	20.93	69.58
34*	F	Elite	22.69	58.00	52.89	45.28	32.57	22.14	76.11
34*	F	Elite	22.76	57.20	54.95	48.57	32.03	23.22	87.94
34*	F	Elite	24.07	58.30	56.16	46.06	32.88	21.07	84.87
34*	F	Elite	26.83	62.30	45.39	44.06			
84*	F	Elite	20.16	58.50	54.57	52.60	33.60	26.00	63.55
84*	F	Elite	21.04	57.60	54.23	50.80	34.22		
84*	F	Elite	21.23	58.00	57.22	53.02	38.87		
84*	F	Elite	21.69	57.60	62.97	57.49	38.54	28.04	77.25
84*	F	Elite	22.18	58.00	62.14	52.23	40.02	28.37	90.06
84*	F	Elite	23.35	63.30	51.70	57.34	35.24		
84*	F	Elite	23.52	61.80	60.88	62.75	39.16		
84*	F	Elite	24.07	61.20	53.98	51.21	38.42	24.55	81.53
84*	F	Elite	24.44	61.20	56.12	52.74	40.32	26.94	87.20
84*	F	Elite	24.94	60.70	53.75	48.11			
84*	F	Elite	25.08	61.00	52.12	47.78	35.59		
84*	F	Elite	25.47	61.20	52.98	50.10	33.21		
84*	F	Elite	26.21	59.80	55.27	56.14	36.91		
84*	F	Elite	26.28	60.20	57.74	54.81	35.14	23.98	75.61
84*	F	Elite	26.54	60.90	62.32	56.33	37.31	26.74	94.77
84*	F	Elite	27.26	61.50	56.07	54.29	36.83	26.21	83.25

Table S1: Raw data of explosive (CMJ, SJ, SL_CMJ) and reactive strength (RSI 1, RSI 2) measurements (*: Athletes of national teams).

89*	F	Elite	18.23	48.10	47.59	43.51	31.55		
89*	F	Elite	18.41	49.20	48.86	45.69	30.24	19.23	93.29
89*	F	Elite	18.52	48.70	50.46	44.01	30.17		
89*	F	Elite	19.15	49.30	45.63	42.49	29.86	16.92	77.26
89*	F	Elite	19.29	49.00	50.48	43.54	31.59	20.70	98.50
89*	F	Elite	19.46	50.00	44.21	42.17	31.62	17.32	77.66
89*	F	Elite	20.23	53.40	45.10	42.76	29.29		
89*	F	Elite	20.30	53.50	51.24	44.82	30.79	18.49	80.85
89*	F	Elite	20.68	53.50	45.97	40.03	28.45	19.98	92.12
89*	F	Elite	20.85	53.20	52.01	44.00	31.53		
89*	F	Elite	21.23	54.90	53.68	47.79	34.26	18.96	71.99
89*	F	Elite	21.39	57.90	46.34	46.93	30.85	17.87	72.49
89*	F	Elite	21.47	57.70	43.40	40.30	30.71	19.13	64.66
89*	F	Elite	22.27	60.30	47.19	45.70	31.19		
89*	F	Elite	22.43	59.80	56.38	46.68	34.83		
89*	F	Elite	22.52	59.60	55.34	45.44	33.58	19.31	72.47
97*	F	Elite	20.35	48.90	47.61	47.62	33.81	22.68	86.09
97*	F	Elite	21.24	50.10	51.46	51.56	36.68		
97*	F	Elite	21.42	51.70	56.84	53.73	39.21		
97*	F	Elite	21.89	49.50	55.26	54.18	35.52	23.25	89.68
97*	F	Elite	22.18	49.80	57.22	56.46	36.36		
97*	F	Elite	22.37	50.30	53.71	52.66	37.91	23.29	104.04
97*	F	Elite	23.58	50.90	50.42	51.58	29.14		
97*	F	Elite	23.75	50.30	57.46	54.11	32.70		
97*	F	Elite	24.26	49.40	59.54	57.74	35.00	23.17	93.10
97*	F	Elite	24.64	50.30	58.16	56.36	31.02	24.35	99.59
122*	F	Elite	18.59	60.80	49.63	49.31	37.26	19.27	48.83
122*	F	Elite	18.88	63.30	51.16	49.93	38.39		
122*	F	Elite	19.07	67.10	50.63	46.31	38.13	18.28	59.50
125*	F	Elite	18.20	58.60	50.45	47.80	34.44	19.32	54.36
127*	F	Elite	18.06	57.60	47.38	40.78	33.85		
127*	F	Elite	18.46	61.20	51.29	44.21	35.13	19.61	63.18
127*	F	Elite	19.49	64.00	43.30	37.54	30.87		
127*	F	Elite	19.69	62.30	46.37	40.68	34.83		
127*	F	Elite	19.86	62.60	45.26	39.15	34.10		
127*	F	Elite	20.08	62.50	46.67	41.96	31.24		
127*	F	Elite	20.10	62.40				20.59	58.65
130*	F	Elite	18.48	70.10	58.62	48.70	35.38	24.58	77.00
130*	F	Elite	18.71	72.30	57.05	48.21	35.93	23.79	78.72
132*	F	Elite	18.14	58.40					
132*	F	Elite	18.29	60.10					
132*	F	Elite	18.83	59.90	57.60	54.41	38.02	23.83	56.81
132*	F	Elite	19.14	56.70	58.13	53.08	35.37		
132*	F	Elite	20.07	57.00	56.50	51.99	37.57		
132*	F	Elite	20.49	58.00	57.78	60.56			
132*	F	Elite	21.14	58.40	61.04	60.62	36.82	25.48	71.17

Table S1: Raw data of explosive (CMJ, SJ, SL_CMJ) and reactive strength (RSI 1, RSI 2) measurements (*: Athletes of national teams).

132*	F	Elite	21.37	58.00	57.34	53.60	33.78	23.53	57.12
154*	F	Elite	18.57	67.10	50.29	45.86			
154*	F	Elite	18.57	67.10	55.53				
154*	F	Elite	18.58	67.20	54.49			19.46	42.37
167*	F	Elite	18.11	53.10	39.95	41.26	27.27		
173*	F	Elite	18.09	60.90	51.93	48.47	29.64		
173*	F	Elite	18.56	58.50	49.40	44.56	27.43	19.43	37.31
173*	F	Elite	18.86	57.50	52.14	49.79	27.88		
173*	F	Elite	19.04	59.00	55.98	47.66	31.68	21.72	70.04
212*	F	Elite	18.04	47.80	48.51	44.34	31.68	23.21	95.41
212*	F	Elite	18.92	42.70	53.49	48.88	36.91	25.12	100.43
212*	F	Elite	19.09	43.60	52.65	48.49	35.27	23.98	93.67
212*	F	Elite	19.93	45.10	48.72	44.96	33.25	23.37	96.08
212*	F	Elite	20.31	46.40	52.06	46.23	33.58	21.95	98.09
212*	F	Elite	20.42	46.60	53.76	49.86	35.55	23.06	98.37
212*	F	Elite	20.87	47.90	51.09	49.97	31.61		
212*	F	Elite	21.03	48.50	47.52	44.75	30.38	20.90	83.85
212*	F	Elite	21.10	48.20	50.75	46.83	31.17		
212*	F	Elite	22.40	51.10	50.74	46.53	33.06	23.15	100.82
212*	F	Elite	23.65	52.90	50.16	44.76	31.37	29.85	110.42
212*	F	Elite	24.07	54.20	53.19	49.07			
212*	F	Elite	24.17	54.00	49.95	50.42			
212*	F	Elite	24.93	57.40	49.56	48.47	31.52	24.27	84.36
212*	F	Elite	25.86	59.70	51.90	47.41			
212*	F	Elite	25.87	59.50	52.50				
212*	F	Elite	25.87	59.60	49.41			21.19	52.76
216*	F	Elite	18.12	57.70	42.22	39.14	29.89	22.65	65.28
216*	F	Elite	18.50	58.90	47.26	40.37	26.43	24.02	68.03
216*	F	Elite	19.06	62.20	48.39	40.54	29.54	21.86	59.43
216*	F	Elite	19.22	61.70	48.04	41.38	29.31	21.03	61.13
216*	F	Elite	19.29	64.30	45.53	38.56	26.95	21.95	60.14
1	F	U14	9.34	30.00	48.25	35.26	23.81		
1	F	U14	10.36	32.40	40.60	40.16	21.79		
4	F	U14	11.15	32.00	45.82	46.40	29.87		
5	F	U14	11.80	38.40	50.87	49.26	26.54		
6	F	U14	11.86	27.90	50.57	43.02	26.20		
8	F	U14	11.88	37.60	47.72	42.83	29.41		
9	F	U14	11.43	26.50	45.90	43.22	26.58		
10	F	U14	11.73	30.30	45.66	45.28	26.16		
12	F	U14	12.85	46.10	47.84	43.16	27.77		
13	F	U14	9.70	25.60	37.00	39.31	21.40	15.12	49.93
13	F	U14	9.93	25.70	38.74	34.19	20.60	14.07	40.12
13	F	U14	10.07	26.70	37.56	34.30	22.16	10.04	34.24
13	F	U14	10.75	28.60	39.42	37.79	21.39	12.03	31.53
13	F	U14	11.87	32.20	40.45	38.36	22.94		
14	F	U14	11.71	32.50	43.11	38.77	24.29		

Table S1: Raw data of explosive (CMJ, SJ, SL_CMJ) and reactive strength (RSI 1, RSI 2) measurements (*: Athletes of national teams).

15	F	U14	11.61	33.40	37.62	37.55	24.35		
16	F	U14	11.45	30.70	39.22	39.31	23.59		
17	F	U14	11.28	30.30	36.48	38.88	26.08		
18*	F	U14	11.16	37.30	46.45	43.50	29.07		
23	F	U14	11.79	33.50	43.83	44.06	28.10		
24*	F	U14	11.06	29.00	49.57	46.55	30.33		
25	F	U14	11.62	31.10	53.02	40.79	31.51		
26*	F	U14	10.91	30.20	50.55	50.37	29.64		
28	F	U14	10.55	30.40	40.37	40.52	25.38	19.28	41.56
28	F	U14	10.72	30.10	39.24	38.18	24.03	19.70	42.74
28	F	U14	11.34	31.70	43.30	40.60	28.20		
35*	F	U14	12.24	32.80	44.56	46.69	31.32		
36*	F	U14	11.77	31.20	44.99	42.19	27.42		
37*	F	U14	11.34	29.00	51.12	52.03	29.55		
38	F	U14	9.32	26.60	38.89	37.39	26.77		
38	F	U14	11.34	33.90	41.03	38.47	25.92		
38	F	U14	12.92	44.00	48.20	46.95	30.36		
38	F	U14	13.39	47.30	51.74	49.14	29.40		
38	F	U14	13.76	50.10	47.85	52.94	31.79	21.04	61.94
39	F	U14	11.36	32.50	44.72	42.45	26.40		
40	F	U14	11.37	30.10	43.72	43.61	30.51		
45	F	U14	11.32	29.30	47.12	46.73	31.45		
47	F	U14	11.14	27.10	49.06	50.02	37.21		
48	F	U14	11.62	34.30	37.00	37.79	21.67	12.87	17.46
48	F	U14	12.20	37.60	33.94	34.60	26.93		
50	F	U14	12.51	41.60	49.31	46.66	32.64	24.96	87.61
51*	F	U14	11.11	31.30	43.90	45.63	26.86		
57	F	U14	10.98	34.60	44.96	44.70	27.83		
60	F	U14	11.39	34.80	43.13	41.64	25.28		
62	F	U14	8.07	27.20	33.74	36.32	23.77	13.45	25.25
62	F	U14	8.30	29.60	31.65	30.39	23.84	13.86	34.91
62	F	U14	8.44	29.40	33.07	31.99	22.32	13.00	36.10
62	F	U14	9.12	32.60	33.09	32.23	21.62		
62	F	U14	11.68	46.50	40.19	36.72	26.74	16.06	37.05
63	F	U14	11.47	37.50	39.73	38.29	28.19		
64	F	U14	10.99	36.20	45.22	45.77	29.80		
65	F	U14	11.09	34.70	44.90	35.67	25.54		
65	F	U14	11.63	37.50	46.83	41.92	27.62		
65	F	U14	12.53	43.10	46.90	35.55	29.14	18.24	44.38
65	F	U14	13.11	48.20	45.60	36.90	25.60		
66	F	U14	11.10	33.70	42.80	45.13	26.16		
67	F	U14	11.48	28.70	33.50	32.80	20.26		
68	F	U14	11.43	33.50	44.76	40.67	27.52		
68	F	U14	12.87	39.00	42.92	40.08	28.68	15.76	34.68
68	F	U14	13.45	43.80	47.57	44.90	32.44		
70	F	U14	12.30	38.70	44.23	41.69	27.31		

Table S1: Raw data of explosive (CMJ, SJ, SL_CMJ) and reactive strength (RSI 1, RSI 2) measurements (*: Athletes of national teams).

70	F	U14	12.77	41.40	44.07	39.61	25.39		
70	F	U14	13.20	44.60	43.55	41.64	26.85	14.78	25.68
73	F	U14	12.66	41.80	44.15	42.53	29.31		
74*	F	U14	12.42	35.10	43.15	40.67	27.23		
76	F	U14	11.29	31.60	41.45	40.65	27.42		
76	F	U14	12.86	38.00	52.95	45.17	32.83		
76	F	U14	13.33	41.30	50.45	47.12	33.25		
76	F	U14	13.70	44.20	45.88	44.25	32.04	21.65	51.21
78	F	U14	11.44	33.10	41.21	39.98	24.99		
79	F	U14	12.12	37.80	52.69	46.45	33.78	24.64	75.01
79	F	U14	12.70	44.60	48.98	45.75	29.19		
81	F	U14	11.77	41.00			28.04		
82	F	U14	11.71	33.40	44.10	41.55	25.96		
82	F	U14	13.28	44.30	42.99	43.75	27.95		
82	F	U14	13.75	50.40	43.14	39.58	25.21		
83	F	U14	10.88	33.90	39.27	34.81	24.63		
83	F	U14	11.89	37.80	37.85	33.61	24.15		
85	F	U14	11.11	31.00	48.44	40.85	29.35		
86	F	U14	12.09	39.40	51.10	48.69	31.68	15.64	41.38
90	F	U14	11.65	31.80	46.10	38.17	26.97		
96	F	U14	11.88	37.80	49.39	44.68	27.42		
100	F	U14	11.49	35.80	48.02	47.00	33.06		
101	F	U14	10.41	26.50	43.78	39.16	27.66	19.01	54.73
101	F	U14	10.64	27.90	42.88	37.60	25.59	18.07	59.69
101	F	U14	10.78	27.20	42.72	39.88	28.71	16.28	53.00
101	F	U14	11.47	29.70	47.73	42.72	32.75	18.51	79.48
101	F	U14	11.55	29.70	49.44	47.13	30.87		
101	F	U14	13.13	34.20	54.14	53.98	36.16		
101	F	U14	13.97	37.10	46.82	50.16	30.87	27.08	82.44
102	F	U14	11.53	37.60	37.47	40.00	30.53		
103	F	U14	10.95	31.70	47.75	41.35	26.38		
104	F	U14	11.25	32.40	41.96	40.75	25.39		
106*	F	U14	11.35	36.20	52.42	47.25	35.60		
106*	F	U14	12.93	44.60	55.62	51.27	34.20		
106*	F	U14	13.39	48.40	60.37	49.93	35.08		
106*	F	U14	13.77	53.10	53.08	52.04	35.06	26.59	92.14
112	F	U14	11.47	37.10	47.78	41.38	27.55		
112	F	U14	13.98	52.20	51.83	45.63	29.14	17.34	49.19
113	F	U14	11.73	35.90	47.16	39.50	24.67	16.22	34.10
113	F	U14	12.31	38.70	44.90	41.17	26.03		
116	F	U14	12.13	41.40	53.93	53.19	35.45	17.44	28.67
116	F	U14	12.71	46.80	55.74	51.92	35.37		
117	F	U14	12.09	35.80	39.68	40.03	23.79		
117	F	U14	12.99	40.20	40.79	40.77	23.77	12.54	15.63
117	F	U14	13.57	47.00	38.59	36.98	22.03		
118	F	U14	11.19	32.00	43.90	43.14	23.80		

Table S1: Raw data of explosive (CMJ, SJ, SL_CMJ) and reactive strength (RSI 1, RSI 2) measurements (*: Athletes of national teams).

120	F	U14	11.72	32.60	42.56	41.09	27.06	0.00	0.00
123	F	U14	11.04	35.00	54.79	52.19	31.09		
124*	F	U14	11.69	36.80	39.06	39.47	26.08		
124*	F	U14	12.71	40.50	41.61	38.86	27.33		
125*	F	U14	10.91	34.20	38.15	37.93	26.52		
126	F	U14	11.05	31.50	43.65	44.13	27.59		
127*	F	U14	11.03	34.00	49.12	43.96	31.45		
129	F	U14	12.41	32.30	51.75	52.35	30.94		
129	F	U14	12.88	34.30	51.39	45.00	26.59		
129	F	U14	13.25	35.80	46.63	47.65	27.35		
130*	F	U14	11.44	37.70	45.42	43.57	26.42		
132*	F	U14	12.08	32.30	55.64	48.15	31.04		
133	F	U14	11.82	37.20	53.18	48.66	30.82		
133	F	U14	13.26	45.00	54.00	48.46	30.69	18.15	23.00
133	F	U14	13.84	50.00	46.62	46.33	29.42		
135	F	U14	11.62	29.90	37.77	38.51	26.88		
136	F	U14	10.98	34.00	44.17	42.49	30.66		
140	F	U14	11.77	41.60	52.70	46.17	37.49		
141*	F	U14	12.00	33.60	39.59	41.36	28.63		
142	F	U14	12.53	30.90	35.28	37.47	26.69		
143	F	U14	10.95	38.20	46.73	40.51	25.73		
143	F	U14	11.49	40.60	47.22	47.98	28.01		
143	F	U14	11.96	45.10	47.67	49.61	32.20		
143	F	U14	12.39	46.60	49.10	49.16	30.73	20.78	43.80
143	F	U14	12.97	48.20	46.82	49.55	30.35		
145	F	U14	11.27	27.70	39.87	40.84	26.40		
146	F	U14	11.70	30.80	44.52	43.03	30.78		
154*	F	U14	11.41	35.50	42.99	41.45	25.42		
157	F	U14	11.10	35.20	43.72	39.38	30.53		
158	F	U14	10.99	33.50	43.83	41.00	26.04		
158	F	U14	12.57	37.60	45.44	42.89	27.92		
158	F	U14	13.03	40.10	44.47	44.40	26.39		
158	F	U14	13.41	41.20	46.45	41.77	29.37	19.61	49.71
160	F	U14	11.02	31.50	35.55	38.97	23.55		
162	F	U14	11.31	33.80	51.95	43.95	29.89		
166	F	U14	10.91	28.30	45.55	41.56	28.47		
168	F	U14	11.29	26.80	41.90	39.46	24.85		
176	F	U14	11.39	30.60	41.56	40.01	23.06		
180	F	U14	9.93	27.50	37.69	37.65	24.71	12.30	57.38
180	F	U14	10.16	28.70	39.18	36.68	25.57	14.01	75.51
180	F	U14	10.29	29.00	38.20	38.90	23.22	13.56	78.65
180	F	U14	10.98	31.90	42.03	39.12	27.05	15.60	64.36
180	F	U14	11.07	31.40	45.01	40.91	29.27		
180	F	U14	12.64	41.60	52.27	46.07	31.52		
180	F	U14	13.48	48.30	46.69	48.37	31.98	23.69	79.55
186	F	U14	11.88	30.80	51.68	47.03	29.72		

Table S1: Raw data of explosive (CMJ, SJ, SL_CMJ) and reactive strength (RSI 1, RSI 2) measurements (*: Athletes of national teams).

194	F	U14	11.89	33.60	42.88	43.10	24.99		
195	F	U14	10.95	40.40	35.94	36.19	21.11		
196	F	U14	10.29	29.80	37.24	34.55	22.17		
196	F	U14	11.30	33.20	45.04	38.83	25.24		
197	F	U14	11.57	34.80	46.78	45.57	30.43		
200	F	U14	11.74	31.50	42.57	45.54	29.01		
201	F	U14	11.19	33.10	43.52	39.02	24.64		
206	F	U14	11.86	38.40	51.34	44.34	26.21		
208	F	U14	11.69	35.70	47.23	39.88	28.12	15.29	32.52
208	F	U14	12.27	40.00	45.74	39.79	28.58		
209	F	U14	10.63	34.60	43.03	41.22	26.77		
211	F	U14	11.68	27.90	45.66	45.06	31.48		
213	F	U14	11.41	33.20	38.95	40.59	28.73		
215	F	U14	11.16	38.30	50.85	46.64	32.09		
223	F	U14	11.43	28.50	44.97	39.50	29.80		
223	F	U14	11.97	30.10	43.03	44.13	27.08		
223	F	U14	13.45	37.40	40.99	40.07	25.38		
224	F	U14	11.08	30.30	44.37	41.86	28.26		
225	F	U14	11.72	32.00	48.42	46.47	29.29		
228	F	U14	11.73	31.50	41.55	45.25	29.44		
229	F	U14	11.74	32.80	33.80	34.78	21.24		
18*	F	U16	15.38	62.60	58.00	53.73	32.86	24.88	67.90
18*	F	U16	15.59	61.70	59.29	53.51	34.69	24.24	61.83
22*	F	U16	14.41	43.00	43.79	41.26	27.68	19.56	66.53
22*	F	U16	15.33	46.10	45.92	42.89	31.25	24.58	81.75
23	F	U16	15.22	54.60	45.95	45.15	29.35	17.96	41.94
24*	F	U16	15.33	50.80	48.14	46.19	33.21	18.47	30.42
24*	F	U16	15.71	51.70	47.09	43.15	32.49	16.09	27.96
24*	F	U16	15.82	52.90	49.03	42.99	33.51	20.26	39.98
26*	F	U16	15.19	49.60	53.57	51.30	31.93	18.12	68.68
26*	F	U16	15.56	52.20	50.84	51.04	31.66	20.05	62.64
26*	F	U16	15.68	53.60	55.07	54.62	33.68	19.49	66.49
36*	F	U16	15.46	51.60	50.37	48.30	32.87	15.77	32.38
37*	F	U16	15.27	48.50	55.93	50.17	31.99	23.75	67.26
37*	F	U16	15.54	48.60	54.79				
37*	F	U16	15.55	48.60	58.64				
37*	F	U16	15.55	48.20	56.77				
37*	F	U16	15.55	48.30	54.72				
37*	F	U16	15.64	48.60	52.04				
37*	F	U16	15.70	50.70	51.76	52.46			
37*	F	U16	15.71	49.60	57.65				
37*	F	U16	15.79	50.50	60.31	52.09			
42*	F	U16	15.84	51.30	44.98	41.83	28.67	22.16	74.57
45	F	U16	14.22	47.30	52.09	49.57	30.76	21.24	55.07
45	F	U16	14.46	50.20	47.06	43.31	27.06	20.76	56.57
45	F	U16	14.59	51.70	62.14	46.56	34.36	17.73	56.77

Table S1: Raw data of explosive (CMJ, SJ, SL_CMJ) and reactive strength (RSI 1, RSI 2) measurements (*: Athletes of national teams).

45	F	U16	15.28	51.90	58.30	50.72	36.31	23.28	75.79
51*	F	U16	14.90	54.20	53.40	42.86	29.43		
51*	F	U16	15.04	54.70	52.94	43.31	30.21	20.57	45.52
51*	F	U16	15.46	55.30	51.52	49.08			
51*	F	U16	15.56	56.80	51.06	46.34	30.09		
63	F	U16	14.99	54.10	50.28	50.36	32.40	19.71	52.85
74*	F	U16	15.69	49.40	45.39	39.08	30.04	17.35	34.13
74*	F	U16	15.86	51.20	43.58	39.81	27.73		
110*	F	U16	14.41	44.70	46.48	44.69	27.69	22.17	76.56
110*	F	U16	15.33	49.60	41.41	42.00	26.86	25.67	82.91
124*	F	U16	14.61	57.00	42.57	40.48	27.41	20.62	79.78
124*	F	U16	14.88	58.80	40.10				
124*	F	U16	14.89	58.60	44.75				
124*	F	U16	14.89	59.00	47.72				
124*	F	U16	14.89	59.10	46.68				
124*	F	U16	14.98	59.00	47.44				
124*	F	U16	15.04	60.80	48.93	40.39			
124*	F	U16	15.13	61.10	41.47	42.50			
125*	F	U16	14.18	51.20	45.85	42.53	28.58	22.21	62.32
125*	F	U16	14.35	50.60	43.58	41.15	29.43	19.77	64.67
125*	F	U16	15.63	57.30	45.24	44.92	32.06	19.14	58.92
127*	F	U16	15.31	56.80	51.07	45.83	36.39	21.33	79.72
127*	F	U16	15.69	59.00	50.25	45.61	31.00	22.67	88.50
127*	F	U16	15.80	59.30	53.25	46.39	32.91	20.44	68.79
132*	F	U16	15.34	49.60	60.06	55.41	34.54	22.27	59.21
132*	F	U16	15.54	51.40	53.96	51.35	32.11	21.57	46.64
132*	F	U16	15.78	52.90					
132*	F	U16	15.86	52.50					
132*	F	U16	15.89	52.90	56.44	51.68	33.54		
136	F	U16	14.50	51.60	52.60	49.26	33.48	23.36	70.43
141*	F	U16	15.27	47.60	52.89	43.71	33.03	23.28	92.17
141*	F	U16	15.44	48.40	57.46	44.76	32.97	24.28	91.74
144*	F	U16	15.27	50.60	48.33	44.62	28.63		
161	F	U16	14.41	45.70	50.26	46.61	29.93		
161	F	U16	15.33	52.80	45.88	43.90	29.56	20.04	56.27
191*	F	U16	15.33	55.10	54.55	46.12	39.33	28.09	71.72
225	F	U16	14.23	46.30	55.12	51.53	33.70	21.12	48.21
18*	F	U18	17.15	63.40	51.66	49.62	32.05		
18*	F	U18	17.65	61.40	54.53	50.21	32.50		
24*	F	U18	16.27	51.60	52.86	49.42	34.00	19.38	48.18
24*	F	U18	16.43	53.40	46.79	43.25	33.31	21.37	41.97
24*	F	U18	16.50	54.70	45.42	43.50	30.96	19.84	50.38
24*	F	U18	17.81	50.30	43.88	41.70	30.93	22.12	83.12
26*	F	U18	16.14	54.10	49.54	51.21	31.49	20.65	80.28
26*	F	U18	16.29	53.50	55.03	54.48	34.71	20.17	93.77
26*	F	U18	16.36	53.60	52.30	53.10	32.60	17.69	75.97

Table S1: Raw data of explosive (CMJ, SJ, SL_CMJ) and reactive strength (RSI 1, RSI 2) measurements (*: Athletes of national teams).

26*	F	U18	17.66	57.20	55.91	55.79	35.65	23.00	102.83
36*	F	U18	16.71	60.30	53.41	50.10	31.68	19.10	59.08
36*	F	U18	16.98	60.60	48.56				
36*	F	U18	16.99	60.10	52.11				
36*	F	U18	16.99	60.40	55.16				
36*	F	U18	17.08	60.50	52.81				
36*	F	U18	17.13	61.40	51.71	53.22			
36*	F	U18	17.14	60.50	54.72				
36*	F	U18	17.23	62.90	48.25	47.46			
37*	F	U18	16.34	53.10	57.54	48.25	32.03	23.23	74.30
37*	F	U18	16.56	52.60	61.12	53.88	35.88	23.46	61.81
42*	F	U18	16.21	53.90	45.70	42.26	28.51	22.73	75.48
42*	F	U18	16.33	54.80	42.24	40.39	30.04	21.30	72.32
51*	F	U18	16.23	58.50	52.07	46.06	28.74		
51*	F	U18	16.34	59.10	53.57	47.83	31.06	20.32	56.70
51*	F	U18	17.25	60.70	53.17	50.95			
51*	F	U18	17.26	60.40	59.71				
51*	F	U18	17.26	60.10	58.18			18.51	34.30
74*	F	U18	16.70	59.10	42.26	39.22	27.92	15.80	45.78
74*	F	U18	17.07	61.10	41.80	36.85	29.93	16.83	47.65
74*	F	U18	17.64	61.40	45.34	40.87	28.85	17.28	58.93
74*	F	U18	17.79	62.50	38.77	33.96	27.56	19.52	59.89
80*	F	U18	16.21	58.70	56.87	50.65	29.64		
80*	F	U18	16.40	59.40	51.73	49.88	28.20	20.75	61.00
89*	F	U18	16.39	47.30	43.31	43.07	27.22	18.44	64.11
89*	F	U18	17.28	48.70	44.35	46.16	28.97		
89*	F	U18	17.46	49.70	44.30	43.62	29.91		
89*	F	U18	17.70	49.60	47.73	46.09	30.35		
89*	F	U18	17.93	50.30	41.89	38.51	27.72	16.44	53.57
89*	F	U18	17.97	49.90	48.87	46.11	31.34		
122*	F	U18	17.05	57.50	50.03	47.34	32.16	18.65	40.53
122*	F	U18	17.94	64.10	54.17	51.26	38.54		
125*	F	U18	16.88	57.80	47.33	46.01	35.46	22.91	79.89
125*	F	U18	17.30	56.90	51.18	48.23			
125*	F	U18	17.40	56.70	54.04	51.10			
127*	F	U18	16.25	58.30	56.38	47.06	35.03	20.89	76.55
127*	F	U18	16.41	58.90	48.75	47.80	33.46	19.71	72.97
127*	F	U18	16.48	57.40	47.26	45.30	33.29	20.51	70.87
127*	F	U18	17.06	59.00	44.19	40.73	29.39		
127*	F	U18	17.26	59.50	49.68	42.08	31.60		
127*	F	U18	17.44	58.50	53.85	43.15	34.43		
127*	F	U18	17.53	57.80	48.50	43.69	34.20	20.16	59.92
127*	F	U18	17.78	58.20	51.12	43.07	33.73	22.12	69.44
130*	F	U18	16.17	65.70	56.91	47.11	33.57	21.60	68.22
130*	F	U18	17.41	68.60	57.05	51.88	32.35	24.19	77.23
130*	F	U18	17.84	70.30	58.49	51.55			

Table S1: Raw data of explosive (CMJ, SJ, SL_CMJ) and reactive strength (RSI 1, RSI 2) measurements (*: Athletes of national teams).

130*	F	U18	17.94	71.60	61.45	53.45			
132*	F	U18	16.73	57.30	54.72	50.52	35.21		
141*	F	U18	16.28	54.70	45.88	42.04	29.04	23.42	87.91
141*	F	U18	16.66	53.60	53.05	43.13	31.84		
141*	F	U18	17.18	52.80	45.63	42.41	27.13		
141*	F	U18	17.37	52.80	43.33	39.15	28.54	20.41	73.23
141*	F	U18	17.45	53.60	49.27	43.85	29.97	19.72	76.28
154*	F	U18	16.35	59.30	51.74	44.46	32.14	22.47	60.92
154*	F	U18	16.78	60.60	48.46	45.61			
154*	F	U18	16.87	61.60	52.79	48.05			
154*	F	U18	17.42	60.70	51.21	42.78	31.85	22.52	73.56
154*	F	U18	17.65	65.70	48.01	43.87	29.71	18.36	60.70
173*	F	U18	16.57	51.90	55.10	46.61	29.71		
173*	F	U18	17.03	53.70	52.25	50.11	31.84	18.77	37.54
173*	F	U18	17.91	58.80	48.80	43.99	29.06		
193	F	U18	17.76	56.70	51.32	51.64	25.90	17.26	30.76
212*	F	U18	16.03	47.80	50.74	48.25	33.24	18.88	74.59
212*	F	U18	16.91	50.00	48.15	45.65	30.26		
212*	F	U18	17.33	52.00	52.82	45.75	31.88		
212*	F	U18	17.56	49.60	53.35	50.62	31.85	21.83	77.87
212*	F	U18	17.86	48.00	55.79	51.02	37.21		
216*	F	U18	16.05	59.50	48.06	42.96	27.88		
216*	F	U18	16.24	58.50	48.31	42.29	27.03	21.96	64.20
216*	F	U18	17.11	59.10	42.48	41.14	27.18	22.85	58.33
216*	F	U18	17.29	57.20	46.83	41.63	29.48	21.94	63.78
19	M	Elite	18.51	73.70	59.53	57.18	32.07	23.96	38.90
19	M	Elite	18.84	73.80	61.02	54.15	33.84		
19	M	Elite	18.86	74.00				24.08	61.57
19	M	Elite	19.71	71.40	60.95	59.61		25.58	70.73
21*	M	Elite	18.57	67.40	68.33	63.35	39.27	25.57	83.72
21*	M	Elite	19.19	67.70	65.31	59.18	36.94	27.47	90.28
21*	M	Elite	19.34	67.20	65.63	62.64	37.08	25.87	91.72
21*	M	Elite	20.40	70.10	67.45	62.85		28.75	84.83
21*	M	Elite	21.61	71.30	63.42	63.42		31.06	101.44
21*	M	Elite	23.24	74.70	66.89	67.15	39.97	32.83	110.00
32*	M	Elite	18.56	60.40	64.68	58.57	39.53	26.94	70.19
32*	M	Elite	19.53	61.50	61.35	55.96	42.24	25.11	71.34
32*	M	Elite	20.00	62.00	64.69	60.13	41.23	27.63	75.83
32*	M	Elite	20.48	62.00	62.49	54.17	40.97	27.89	79.55
32*	M	Elite	21.51	63.00	64.99	52.91	41.18	28.22	76.65
32*	M	Elite	22.41	64.00	65.98	55.81			
32*	M	Elite	23.50	64.00	68.02	58.85	40.07	28.66	83.69
32*	M	Elite	25.38	64.10	61.14	56.73		26.62	83.47
32*	M	Elite	26.57	64.40	59.12	55.32			
41*	M	Elite	19.32	57.90	69.09	59.77	40.75	26.26	68.35
41*	M	Elite	19.65	59.10	69.92	63.05	41.85	29.10	69.38

Table S1: Raw data of explosive (CMJ, SJ, SL_CMJ) and reactive strength (RSI 1, RSI 2) measurements (*: Athletes of national teams).

41*	M	Elite	20.27	58.90	71.15	58.62	41.16	28.38	66.28
41*	M	Elite	20.74	60.20	71.29	60.26	43.70	30.19	67.67
41*	M	Elite	21.22	60.70	67.91	62.15	42.96	30.48	76.87
41*	M	Elite	22.25	62.20	71.53	60.03	43.60	30.95	70.87
41*	M	Elite	23.15	63.10	70.05	56.81			
41*	M	Elite	24.24	62.60	68.51	61.18	44.16	32.08	86.41
41*	M	Elite	26.10	62.30	66.69	58.82			
41*	M	Elite	26.12	62.90				29.13	77.29
41*	M	Elite	27.37	64.60	69.11	57.26		29.64	80.22
44*	M	Elite	26.56	66.50	67.27	57.96	39.14	23.44	84.85
44*	M	Elite	27.41	68.10	62.15	59.74	40.51	23.88	59.90
44*	M	Elite	27.60	69.00	63.67	55.76	41.14	22.69	49.75
44*	M	Elite	28.04	67.90	73.96	56.77	41.67	22.74	52.11
44*	M	Elite	28.66	69.10	66.13	56.93	40.87	24.08	49.95
44*	M	Elite	29.13	68.30	67.98	56.11	40.84	23.92	50.75
44*	M	Elite	29.58	67.90	64.37	54.96	40.43	22.47	60.04
44*	M	Elite	29.72	68.70	63.24	59.38	42.99	24.58	65.36
44*	M	Elite	29.83	66.70	62.05	56.24	40.91	24.56	81.96
53	M	Elite	19.15	61.30	57.33	59.68	37.56		
53	M	Elite	19.71	61.50	68.21	58.26	38.63	26.78	70.84
53	M	Elite	21.15	64.80	58.02	57.11	37.70	25.89	67.44
54	M	Elite	18.05	58.70	55.80	57.13	36.19		
54	M	Elite	18.13	59.00	60.24	54.02	34.80		
54	M	Elite	18.54	61.10	55.60	51.49	35.07		
54	M	Elite	19.28	64.60	57.55	53.24		25.66	77.36
58*	M	Elite	19.08	67.20	52.74	51.57	34.15	21.57	66.57
58*	M	Elite	19.66	67.10	56.68	46.81	30.67	21.15	70.29
58*	M	Elite	19.74	66.10	56.55	48.77	31.85	21.55	74.91
58*	M	Elite	20.87	68.00	54.71	49.04		22.03	71.62
59*	M	Elite	19.15	60.30	84.22	67.01	41.68		
59*	M	Elite	21.14	65.40	77.98	66.71	44.66	28.73	61.58
59*	M	Elite	21.63	66.10	73.37	66.23	39.39	26.08	62.44
59*	M	Elite	22.66	65.60	71.23	62.69	42.22		
59*	M	Elite	22.71	65.50				22.28	64.32
59*	M	Elite	23.55	67.30	76.64	70.88			
59*	M	Elite	27.50	66.10	67.51	63.15			
69	M	Elite	18.89	66.60	56.67	53.00	36.36	26.79	79.80
72	M	Elite	18.26	61.80	62.84	52.31	38.37	18.83	69.76
72	M	Elite	20.26	67.10	57.01	49.57	38.94	28.46	93.69
77*	M	Elite	19.11	69.50	59.94	52.57	35.51	26.60	78.92
87*	M	Elite	18.43	65.40	59.38	53.99	35.28		
87*	M	Elite	18.45	65.80				28.20	88.39
87*	M	Elite	22.13	69.10	59.02	48.91	32.76	23.95	65.78
87*	M	Elite	23.27	68.40	60.39	51.61		27.25	76.25
87*	M	Elite	23.48	69.90	55.30	50.37	31.29		
87*	M	Elite	24.13	68.80	51.87	49.55	29.99		

Table S1: Raw data of explosive (CMJ, SJ, SL_CMJ) and reactive strength (RSI 1, RSI 2) measurements (*: Athletes of national teams).

88*	M	Elite	18.36	64.10	59.59	60.27	36.87	21.19	58.02
88*	M	Elite	18.44	63.70	58.78	57.68	37.19	18.80	55.40
88*	M	Elite	19.49	65.70	63.30	58.66	39.58		
94	M	Elite	19.49	72.80	55.77	58.16	34.20		
99	M	Elite	18.10	58.80	51.12	49.70	33.11		
99	M	Elite	18.79	62.90	52.27	54.68	35.59	25.01	82.46
108*	M	Elite	23.21	66.90	49.86	48.09	30.23	20.68	72.23
108*	M	Elite	24.08	64.40	50.08	48.09	30.07	20.88	46.48
108*	M	Elite	24.25	66.80	59.95	54.74	36.02	22.62	55.02
108*	M	Elite	24.69	66.20	60.11	51.45	33.79	23.39	59.43
108*	M	Elite	25.31	66.10	50.58	52.17	31.18	23.12	57.26
108*	M	Elite	25.78	67.50	52.78	51.95	31.84	24.11	60.17
108*	M	Elite	26.27	67.10	53.07	50.94	32.61	24.03	57.09
137*	M	Elite	19.09	64.30	61.91	58.00		22.27	67.88
138*	M	Elite	19.13	64.60	53.69	55.78		28.97	111.67
139*	M	Elite	24.53	59.80	40.30	38.75	24.95		
139*	M	Elite	24.82	66.80	50.86	42.74	29.19		
139*	M	Elite	25.29	66.60	45.53	46.94	28.60		
147	M	Elite	19.23	60.90	53.16	51.45	31.60	22.40	72.30
147	M	Elite	27.99	65.80	46.84	46.13	29.59		
151*	M	Elite	18.11	72.00	61.32	54.56	37.44	25.71	59.12
151*	M	Elite	19.56	74.20	57.28	50.56	38.43	23.64	65.49
151*	M	Elite	20.20	74.30	61.76	55.61			
151*	M	Elite	21.25	75.20	60.71	54.95	36.70		
151*	M	Elite	21.50	74.00	63.80	59.69	43.28	26.22	70.99
151*	M	Elite	23.37	75.60	63.06	55.16		25.80	69.58
151*	M	Elite	24.63	76.20	60.71	54.47		25.78	68.77
151*	M	Elite	25.54	76.40	56.26	51.40	36.03		
152*	M	Elite	21.35	66.10	61.38	60.11	38.30		
152*	M	Elite	21.92	67.20	65.23	61.16	42.08	24.90	57.28
152*	M	Elite	24.92	71.50	66.14	62.15	43.27	25.72	83.74
152*	M	Elite	26.66	69.90	66.57	64.14			
152*	M	Elite	26.68	69.60	66.26	60.54			
152*	M	Elite	26.70	72.00	64.02	57.38			
152*	M	Elite	26.74	70.60	66.24	61.78			
152*	M	Elite	26.76	70.00	65.86	62.41			
152*	M	Elite	26.82	71.50	62.96	66.34			
152*	M	Elite	26.84	70.90	62.72	60.45	40.59	25.32	100.52
152*	M	Elite	26.86	72.00	62.82	58.36			
152*	M	Elite	26.88	71.60	66.58	61.04			
152*	M	Elite	26.90	70.90	63.10	60.31			
152*	M	Elite	26.92	72.40	64.70	61.19			
152*	M	Elite	30.66	67.90				30.22	81.23
153*	M	Elite	19.69	76.70	62.19	55.12	38.12	22.89	42.47
153*	M	Elite	19.89	73.00	64.11	57.27	38.78	20.52	48.87
153*	M	Elite	20.33	75.70	66.78	58.19	40.29	23.32	51.89

Table S1: Raw data of explosive (CMJ, SJ, SL_CMJ) and reactive strength (RSI 1, RSI 2) measurements (*: Athletes of national teams).

153*	M	Elite	20.95	77.30	64.02	57.28	38.71	22.68	47.52
153*	M	Elite	21.42	75.00	65.29	55.53	39.29	22.77	48.99
153*	M	Elite	21.90	75.20	67.00	56.30	39.71	24.60	53.04
153*	M	Elite	22.93	76.00	68.35	56.52	44.72	25.00	59.04
153*	M	Elite	23.62	74.60	65.27	58.50			
155	M	Elite	18.40	64.30	60.34	55.95	34.01	27.09	74.15
159*	M	Elite	21.44	64.50	58.08	56.87	34.80		
163*	M	Elite	22.01	63.20	68.01	59.00	38.17	28.55	69.17
163*	M	Elite	22.21	61.90	63.79	60.94	42.31	29.50	85.75
163*	M	Elite	22.65	62.90	61.39	57.29	41.96	29.16	80.89
163*	M	Elite	23.27	63.60	65.06	58.16	40.17	30.69	86.72
163*	M	Elite	23.74	63.30	66.80	60.96	44.37	27.83	84.67
163*	M	Elite	24.22	63.10	65.08	60.05	42.81	29.68	83.79
163*	M	Elite	31.19	70.00	55.67	49.64			
165*	M	Elite	19.86	70.00	54.55	43.13			
165*	M	Elite	21.16	72.40	55.04	50.23	34.78	21.25	57.66
165*	M	Elite	21.82	70.20	59.04	53.04	33.65	20.02	55.19
165*	M	Elite	21.90	73.90	55.14	50.30	30.63	20.25	51.39
165*	M	Elite	24.23	74.70	50.92	48.12		21.69	59.10
175*	M	Elite	24.46	67.00	59.71	54.73	34.96	21.02	47.03
175*	M	Elite	25.52	68.90	57.95	53.47	33.44	20.92	47.69
175*	M	Elite	25.61	70.30	48.72	46.69			
177	M	Elite	20.66	64.50	63.84	51.18		22.29	60.42
179*	M	Elite	18.52	51.00	53.80	47.51	33.05	20.63	58.32
179*	M	Elite	19.13	55.30	56.00	51.30			
179*	M	Elite	19.40	54.80	56.62	50.92		18.61	63.33
179*	M	Elite	20.51	58.40	59.56	55.01	33.70	24.65	83.46
179*	M	Elite	22.38	59.30	56.73	52.20		22.96	66.86
179*	M	Elite	22.59	59.60	52.94	48.42	31.93		
179*	M	Elite	23.38	60.10	56.48	50.77		27.95	83.44
179*	M	Elite	23.58	60.10	59.02	55.33		26.09	71.30
182*	M	Elite	20.21	61.80	69.93	58.54	42.57	21.31	65.42
182*	M	Elite	20.78	62.50	57.79	58.60	37.77		
182*	M	Elite	21.05	62.10	62.45	63.54	40.16	23.18	57.43
182*	M	Elite	21.25	62.30	67.37	64.19	39.01	23.63	56.12
182*	M	Elite	21.69	62.40	62.74	57.53	40.44	25.84	58.30
182*	M	Elite	22.31	63.40	70.70	55.83	39.93	26.13	61.67
182*	M	Elite	22.78	63.30	62.96	66.56	42.29	28.09	69.93
182*	M	Elite	23.26	63.70	64.61	63.32	42.51	28.11	71.20
182*	M	Elite	24.29	63.40	65.27	57.23	44.57	25.39	64.57
182*	M	Elite	25.19	63.10	67.39	56.63			
182*	M	Elite	26.28	62.80	67.09	58.80	43.37	25.20	78.31
184*	M	Elite	20.52	64.30	54.75	50.82	31.28		
184*	M	Elite	20.72	65.00	56.49	50.23	33.89		
184*	M	Elite	21.02	65.50	54.72	54.96	33.07		
184*	M	Elite	21.29	65.90	54.79	53.33	35.68	24.79	55.75

Table S1: Raw data of explosive (CMJ, SJ, SL_CMJ) and reactive strength (RSI 1, RSI 2) measurements (*: Athletes of national teams).

184*	M	Elite	21.48	65.20	57.96	53.23	35.06	22.12	71.26
184*	M	Elite	22.54	65.60	59.35	55.96	36.44	24.39	62.07
184*	M	Elite	23.01	65.70	56.51	58.41	37.21	26.28	67.20
184*	M	Elite	23.50	65.80	59.65	56.16	38.62	26.53	75.90
184*	M	Elite	24.53	65.50	58.42	52.47	39.74		
184*	M	Elite	24.56	65.20				27.23	72.22
184*	M	Elite	25.81	65.50	58.50	52.92	34.81		
184*	M	Elite	26.51	65.90	62.47	57.34	38.42	25.54	76.07
184*	M	Elite	26.88	66.10	60.49	57.50	34.35		
184*	M	Elite	28.38	67.90	62.49	57.65		24.77	75.60
185*	M	Elite	23.48	63.40	69.51	58.26	38.25	24.23	60.84
185*	M	Elite	24.52	60.80	73.36	55.40	39.43	26.00	62.88
185*	M	Elite	24.96	62.70	76.97	63.82	43.14	25.06	56.56
185*	M	Elite	25.58	63.80	76.36	60.74	37.99	29.05	66.91
185*	M	Elite	26.05	64.30	69.62	60.05	37.52	23.72	30.36
185*	M	Elite	26.53	65.30	70.13	58.26	35.85	22.12	57.42
189*	M	Elite	19.00	59.90	63.47	59.02	35.90	22.44	71.35
189*	M	Elite	20.04	62.40	67.46	62.21	32.56	23.12	55.42
189*	M	Elite	20.48	63.20	64.36	57.77	33.52	22.52	58.33
189*	M	Elite	21.10	65.10	63.72	59.92	30.06	21.13	53.75
189*	M	Elite	21.36	65.00	61.81	59.08	30.40		
189*	M	Elite	21.57	63.80	62.55	59.83	32.75	25.77	64.58
189*	M	Elite	22.05	66.00	62.78	55.41	33.39	23.79	60.23
190*	M	Elite	18.07	60.60	52.82	46.14	38.83	24.83	70.56
190*	M	Elite	18.89	62.00	57.31	51.08		25.45	74.96
190*	M	Elite	19.98	64.50	52.21	48.11		23.79	74.36
190*	M	Elite	20.23	66.90	55.71	52.10		24.02	72.21
190*	M	Elite	20.89	62.90	51.75	49.60			
190*	M	Elite	21.07	68.50	48.49	43.98	31.25		
190*	M	Elite	21.87	67.30	54.75	45.80		25.53	69.75
190*	M	Elite	22.51	66.60	45.28	42.33	29.40		
190*	M	Elite	22.70	67.70					
190*	M	Elite	22.78	65.90	44.90	43.05	29.02		
190*	M	Elite	22.97	65.40	46.33	43.20			
190*	M	Elite	24.81	71.50					
198*	M	Elite	18.48	63.40	59.12	52.01	39.96	30.97	75.34
198*	M	Elite	20.63	64.00	50.66	52.10	37.06	25.45	86.08
198*	M	Elite	20.92	64.60	55.11	49.96	39.44	21.59	59.94
198*	M	Elite	21.81	65.70	62.03	61.12		26.79	87.09
198*	M	Elite	22.90	66.60	64.56	59.66	41.00	25.27	72.49
198*	M	Elite	24.79	65.90	57.96	53.26		25.99	84.20
199*	M	Elite	20.76	62.10	60.74	50.47	35.52	22.86	62.90
199*	M	Elite	21.80	60.80	64.35	61.53	36.35	25.57	64.33
199*	M	Elite	22.24	62.20	64.46	57.36	36.97	24.06	72.89
199*	M	Elite	22.86	65.40	62.58	59.48	42.18	26.11	68.66
199*	M	Elite	23.33	64.00	64.18	63.49	38.61	29.21	74.03

Table S1: Raw data of explosive (CMJ, SJ, SL_CMJ) and reactive strength (RSI 1, RSI 2) measurements (*: Athletes of national teams).

207*	M	Elite	22.39	64.50	63.12	54.90	39.31	18.99	54.01
207*	M	Elite	23.26	64.70	62.05	53.33	37.07	24.18	47.77
207*	M	Elite	23.43	65.40	65.52	55.32	37.63	24.23	52.93
207*	M	Elite	24.49	66.50	58.13	53.13	34.67	22.59	35.75
214*	M	Elite	18.55	69.60	59.89	51.24	35.19		
214*	M	Elite	18.76	69.10	57.84	52.59	36.67	22.06	46.99
214*	M	Elite	18.95	70.30	64.11	59.07	38.45	23.14	55.52
214*	M	Elite	19.12	71.20				20.38	27.53
214*	M	Elite	19.39	70.90	69.42	54.31	37.78	24.18	47.17
214*	M	Elite	20.03	70.50	69.56	58.04	38.68		
214*	M	Elite	20.48	72.80	67.26	56.43	40.97	26.24	63.58
214*	M	Elite	20.97	74.20	67.35	53.49	43.60	24.83	56.05
214*	M	Elite	22.00	75.20	74.53	57.06	42.38	23.84	61.66
214*	M	Elite	22.89	76.80	66.71	61.10			
214*	M	Elite	23.98	79.20	67.94	59.67	41.42	27.74	78.91
214*	M	Elite	25.76	72.40	67.05	57.31	42.72	27.41	79.50
220*	M	Elite	19.04	71.40	55.19	53.31	35.83	23.77	74.50
220*	M	Elite	20.84	76.10	58.19	54.72		22.08	60.42
220*	M	Elite	21.51	74.80	55.57	53.23		26.28	88.91
220*	M	Elite	22.03	76.00	56.43	53.40		26.26	82.17
220*	M	Elite	22.46	77.90	54.75	50.06	32.95		
2	M	U14	13.34	49.00	47.29	45.44	27.01		
7	M	U14	13.38	35.10	41.06	38.03	24.68		
11	M	U14	13.89	44.70	44.99	42.31	25.03		
20	M	U14	12.44	32.50	44.57	42.89	31.12		
20	M	U14	13.48	37.50	51.51	43.65	30.26		
27	M	U14	13.89	41.10	41.61	41.30	25.02		
29	M	U14	12.31	38.30	38.24	34.77	27.26		
29	M	U14	13.32	40.10	39.30	36.72	26.10		
30	M	U14	13.51	37.30	48.55	43.38	28.27		
31	M	U14	13.45	38.60	42.67	43.87	27.95		
33	M	U14	11.83	35.90	43.88	38.86	25.49		
43	M	U14	11.41	33.40	41.48	41.76	29.02		
46	M	U14	11.43	27.60	43.45	42.78	25.82		
49	M	U14	12.13	40.60	37.93	33.80	23.14		
52	M	U14	13.81	33.90	44.51	43.83	29.59		
54	M	U14	12.00	32.20	43.90	41.03	26.40		
55	M	U14	11.02	32.00	44.47	40.67	27.47		
61	M	U14	13.27	41.00	40.38	35.97	25.52		
77*	M	U14	13.71	41.90	46.33	43.43	30.55		
88*	M	U14	12.31	34.30	52.51	52.77	35.56		
88*	M	U14	13.32	41.40	52.35	51.96	30.03		
91	M	U14	13.38	44.00	42.30	36.01	21.13		
92*	M	U14	13.89	42.90	45.46	47.06	27.60		
93	M	U14	12.69	43.00	42.55	38.23	27.24		
93	M	U14	13.72	47.60	44.06	39.58	26.57		

Table S1: Raw data of explosive (CMJ, SJ, SL_CMJ) and reactive strength (RSI 1, RSI 2) measurements (*: Athletes of national teams).

98	M	U14	13.61	44.60	43.40	38.62	28.54		
105	M	U14	13.80	42.20	45.72	46.53	30.37		
109	M	U14	13.43	44.10	40.29	40.04	24.74		
111*	M	U14	11.55	36.30	40.44	41.69	28.10		
111*	M	U14	12.58	39.70	40.47	41.77	28.47		
114	M	U14	13.36	42.70	39.01	40.81	24.73		
121	M	U14	12.63	32.90	41.01	37.18	25.97		
128	M	U14	12.38	33.10	43.30	42.25	24.66		
131	M	U14	13.08	32.90	40.60	39.28	25.62		
134	M	U14	13.07	46.80	46.33	38.52	28.19		
137*	M	U14	13.69	34.40	40.85	35.51	27.43		
138*	M	U14	13.72	38.90	43.67	44.03	31.97		
149	M	U14	13.90	38.30	51.17	45.20	30.34		
156	M	U14	13.51	47.40	42.26	39.52	25.48		
164	M	U14	13.34	37.00	49.67	46.56	29.88		
170	M	U14	13.46	51.40	51.04	46.63	34.08		
172	M	U14	11.41	32.30	36.35	36.06	24.27		
174	M	U14	13.13	42.50	54.13	56.69	33.26		
181	M	U14	13.91	41.90	38.96	34.87	26.97		
183	M	U14	12.87	37.30	40.57	40.54	24.72		
188	M	U14	12.19	34.40	38.65	40.90	25.17		
192	M	U14	13.19	29.30	37.75	38.08	24.80		
202	M	U14	12.84	39.70	46.07	37.07	27.10		
204	M	U14	13.25	41.40	46.78	43.13	27.50		
205	M	U14	11.65	33.30	42.65	40.06	24.95		
210	M	U14	13.66	37.00	40.75	40.48	25.64		
217	M	U14	12.52	39.90	48.08	43.93	26.85		
218	M	U14	12.21	39.90	41.88	39.44	26.73		
219	M	U14	11.10	28.80	39.76	36.04	26.41		
219	M	U14	12.13	31.40	44.85	41.32	32.32		
221	M	U14	13.94	36.00	53.00	47.67	30.87		
226	M	U14	11.34	29.60	45.48	45.98	28.27		
19	M	U16	15.44	64.60	54.82	46.44	30.62		
19	M	U16	15.78	64.10	61.42	53.03	32.47	20.35	34.23
54	M	U16	15.46	43.30	47.31	45.66	30.89	23.38	79.96
56	M	U16	15.40	53.40	49.73	47.38	29.64		
71	M	U16	15.02	60.90	65.91	57.33	36.65	22.13	51.27
71	M	U16	15.47	63.10	57.91	55.16	35.43	17.87	41.69
75	M	U16	14.61	55.80	49.79	42.98	27.27		
77*	M	U16	14.71	51.80	50.08	49.02	30.27		
77*	M	U16	15.73	61.40	52.90	55.05	34.04		
87*	M	U16	14.34	40.70	45.07	41.33	23.97	9.94	23.58
87*	M	U16	15.37	49.00	53.75	49.76	31.17	15.59	31.28
87*	M	U16	15.99	53.80	55.92	48.30	35.26	24.09	54.95
88*	M	U16	15.77	56.20	60.62	56.38	37.74	19.64	39.99
99	M	U16	15.74	52.50	47.32	44.18	31.23	19.95	61.35

Table S1: Raw data of explosive (CMJ, SJ, SL_CMJ) and reactive strength (RSI 1, RSI 2) measurements (*: Athletes of national teams).

105	M	U16	15.24	51.50	46.43	45.53	28.33	12.90	46.61
107	M	U16	14.94	54.90	45.13	43.35	26.21		
115	M	U16	15.60	54.90	52.27	42.96	25.61	18.90	37.58
115	M	U16	15.64	55.20	45.22	44.10	25.36		
119	M	U16	14.57	57.10	54.76	48.50	35.04		
119	M	U16	15.60	64.80	54.34	50.54	34.92		
131	M	U16	14.11	35.00	43.93	41.71	27.14		
137*	M	U16	14.70	40.50	51.75	48.81	34.97		
137*	M	U16	15.71	48.00	55.09	56.25	33.56	0.00	0.00
138*	M	U16	14.73	42.70	47.66	45.16	30.41		
138*	M	U16	15.74	49.90	55.07	45.86	32.63		
148	M	U16	15.61	57.50	51.54	45.84	29.52	17.61	32.69
148	M	U16	15.65	57.50	50.30	45.91	31.81		
149	M	U16	15.34	49.50	57.63	47.96	34.44	18.23	54.09
155	M	U16	15.09	41.10	55.32	48.14	30.54	22.02	54.22
155	M	U16	15.54	44.40	54.24	50.29	31.64	19.31	49.92
159*	M	U16	14.43	34.90	40.01	39.77	30.73		
165*	M	U16	15.35	48.50	43.54	37.88	24.53	15.25	42.03
165*	M	U16	15.77	53.10	41.68	39.49	24.98		
169	M	U16	14.76	46.80	46.52	42.19	28.69		
169	M	U16	15.78	53.10	53.68	47.05	29.04		
171	M	U16	15.55	45.40	54.44	43.96	32.30		
171	M	U16	15.96	49.00	47.12	46.82	30.26	22.09	63.35
178	M	U16	14.84	46.30	51.24	42.78	31.45		
179*	M	U16	15.56	39.30	46.73	41.40	28.98	12.49	31.64
183	M	U16	14.91	53.90	49.94	47.96	30.83		
187	M	U16	14.19	51.90	49.81	41.51	27.49		
188	M	U16	14.23	41.90	42.80	33.83	23.65		
190*	M	U16	14.96	52.00	49.16	45.23	26.29	16.68	31.57
190*	M	U16	15.58	54.10	47.32	47.44	33.21	19.80	48.83
192	M	U16	14.18	43.20	52.71	48.17	30.28		
192	M	U16	15.19	51.80	53.52	48.25	32.86		
192	M	U16	15.59	56.70	53.13	50.68	31.49	17.07	55.97
192	M	U16	14.15	33.10	39.96	35.71	22.73	17.85	72.30
203	M	U16	14.66	52.30	55.96	49.05	34.01		
203	M	U16	15.01	55.30	60.91	55.41	36.93	18.77	44.50
203	M	U16	15.63	57.90	65.98	60.73	36.38	25.53	44.72
220*	M	U16	14.54	42.90	50.68	45.86	28.00	18.02	58.98
227	M	U16	15.53	62.40	46.05	45.05	29.08		
3	M	U18	18.51	67.30	61.03	60.51	34.80	24.72	52.45
19	M	U18	16.40	67.90	64.45	54.95	33.89	21.37	41.77
19	M	U18	16.85	71.00	59.44	54.52	31.56	21.92	44.47
19	M	U18	17.43	71.00	62.58	57.38	34.18	27.95	37.81
19	M	U18	17.81	70.70	61.87	63.69	33.52	26.04	56.15
21*	M	U18	16.61	61.00	62.25	58.01	33.71	25.97	88.61
54	M	U18	17.47	55.20	52.68	51.03	33.62	24.50	72.22

Table S1: Raw data of explosive (CMJ, SJ, SL_CMJ) and reactive strength (RSI 1, RSI 2) measurements (*: Athletes of national teams).

58*	M	U18	17.07	61.50	52.51	49.68	32.81	15.38	62.43
69	M	U18	16.89	61.00	58.24	50.93	35.72	23.60	68.87
71	M	U18	16.05	65.30	64.92	58.97	35.65	19.20	50.95
71	M	U18	16.43	66.10	65.13	63.02	34.55	27.21	59.37
87*	M	U18	16.44	57.00	54.20	47.58	31.40	21.96	48.85
87*	M	U18	17.02	60.60	57.46	55.03	34.45	21.90	38.63
87*	M	U18	17.39	62.40	55.71	52.18	34.52	24.62	63.32
88*	M	U18	17.78	61.30	57.13	54.90	35.84	22.12	63.10
92*	M	U18	17.95	67.20	52.55	53.20	32.10		
95	M	U18	17.00	55.20	74.90	70.81	38.53	23.62	75.80
99	M	U18	16.43	55.10	47.99	46.04	32.08	22.72	73.40
99	M	U18	16.66	55.70	50.89	48.38	33.33	22.75	69.77
99	M	U18	16.77	56.90	54.05	51.16	33.56	24.19	86.22
99	M	U18	17.39	58.30	57.57	57.07	35.75	27.25	87.60
115	M	U18	16.05	59.10	46.34	42.26	28.93	18.61	55.40
115	M	U18	16.63	62.60	52.48	47.14	31.06	23.56	64.46
115	M	U18	18.10	68.90	50.51	47.41	30.50	26.64	75.98
150	M	U18	17.18	68.80	71.85	61.37	38.14	26.97	73.31
150	M	U18	17.79	71.90	75.96	66.38	41.84	24.32	46.50
151*	M	U18	16.46	66.30	54.92	49.88	34.52	21.22	53.13
151*	M	U18	17.08	68.90	55.51	53.71	38.03	22.33	53.60
151*	M	U18	17.53	69.70	57.70	51.23	35.45	20.88	45.15
155	M	U18	16.12	47.90	53.42	50.73	29.40	21.99	54.95
155	M	U18	16.49	52.50	58.03	52.23	32.38	22.11	57.48
155	M	U18	17.59	60.20	57.47	55.44	32.58	26.01	69.18
165*	M	U18	16.12	55.60	42.39	38.66	25.82		
165*	M	U18	16.73	61.00	44.04	42.49	27.40	19.06	41.20
165*	M	U18	17.19	64.50	42.31	40.66	27.73	14.74	41.79
165*	M	U18	17.76	65.50	48.76	46.86	29.72	18.19	41.34
179*	M	U18	16.08	42.50	48.70	45.70	29.38	17.90	45.28
179*	M	U18	16.54	46.30	53.02	49.36	30.01	18.71	41.97
179*	M	U18	16.91	47.60	53.70	49.03	29.81		
179*	M	U18	17.11	48.70	52.50	46.98	29.74	19.39	64.15
179*	M	U18	17.49	50.80	57.19	51.12	31.22	19.51	60.17
190*	M	U18	16.03	57.80	49.31	42.61	33.39	21.89	56.31
190*	M	U18	16.98	61.80	53.21	47.96	35.45	23.03	57.09
192	M	U18	16.17	58.20	58.60	55.57	33.89	21.48	61.59
192	M	U18	17.64	63.90	59.43	56.39	35.22	23.88	75.98
198*	M	U18	17.86	59.80	58.93	53.43	39.14	26.87	83.23
203	M	U18	16.08	60.20	62.68	57.16	37.23	23.15	54.03
203	M	U18	16.66	60.90	58.78	54.88	36.48	25.18	62.20
220*	M	U18	17.03	61.50	54.33	48.75	34.24	20.73	71.89
222	M	U18	16.82	59.80	57.56	53.74	32.45	22.76	50.49
222	M	U18	17.27	62.90	52.28	48.91	34.00	21.37	52.81
222	M	U18	17.85	66.40	55.95	53.58	32.75	23.84	51.94
222	M	U18	18.23	68.20	55.70	55.45	33.84	22.84	54.58

Table S1: Raw data of explosive (CMJ, SJ, SL_CMJ) and reactive strength (RSI 1, RSI 2) measurements (*: Athletes of national teams).
